# Analysis of controlling genes for tiller growth of *Psathyrostachys juncea* based on transcriptome sequencing technology

**DOI:** 10.1186/s12870-022-03837-w

**Published:** 2022-09-23

**Authors:** Zhen Li, Lan Yun, Xiaomin Ren, Fengling Shi, Fugui Mi

**Affiliations:** 1 College of Grassland, Resources and Environment, Inner Mongolia Agricultural University, Hohhot, 010018 China; 2 Key Laboratory of Grassland Resources of the Ministry of Education and Key Laboratory of Forage Cultivation, Processing and High Efficient Utilization of the Ministry of Agriculture, Hohhot, 010018 China

**Keywords:** *Psathyrostachys juncea*, Tillering regulation, Transcriptome, Plant hormones

## Abstract

**Background:**

Tillering is a complicated process in plant and is a significant trait that affects biomass and seed yield of bunch grass *Psathyrostachys juncea*, a typical perennial forage species. To clarify the regulatory mechanisms of tillering in *P. juncea* and to explore related candidate genes could be helpful to improve the seed and forage yield of perennial gramineous forages. We selected the tiller node tissues of *P. juncea* for transcriptome sequencing to determine the differentially expressed genes (DEG) between dense and sparse tillering genotypes. The metabolic pathway was studied, candidate genes were screened, and reference genes stability were evaluated.

**Results:**

The results showed that approximately 5466 DEGs were identified between the two genotypes with dense and sparse tillers of *P. juncea*, which significantly differed in tiller number. Tillering regulation pathways analysis suggested that DEGs closely related to the biosynthesis of three plant hormones, namely auxin (IAA), cytokinin (CTK), and strigolactones (SLs), while “biosynthesis of lignin” and “nitrogen metabolism” have remarkable differences between the dense and sparse tillering genotypes. Meanwhile, the reference gene *Actin1*, having the best stability, was screened from twelve genes with highest expression level and was used in verification of ten tillering related candidate genes.

**Conclusions:**

The tillering mechanism of perennial grass *P. juncea* was expounded by transcriptome analysis of tiller node tissues. We demonstrated that dense-tillering genotypes may be distinguished by their low expression patterns of genes involved in SL, IAA, and high expression patterns of genes involved in CTK biosynthesis at the tillering stage, and nitrogen metabolism and lignin biosynthesis can also affect the number of tillers. Furthermore, the expression level of ten tillering related candidate genes were verified using *Actin1* as reference gene. These candidate genes provide valuable breeding resources for marker assisted selection and yield traits improvement of *P. juncea*.

**Supplementary Information:**

The online version contains supplementary material available at 10.1186/s12870-022-03837-w.

## Background

*Psathyrostachys juncea* is a forage species of Gramineae, *Triticeae*, with diploid chromosome level (2n = 2x = 14) and is a cross-pollinated, perennial bunchgrass. Wild *P. juncea* germplasm originated in central and northern Asia’s inland areas and their distribution has extended to East Asia, Europe and North America due to domestication and introduction [1]. Wild plants of *Psathyrostachys* are mainly distributed in the north of the Tianshan Mountains in Xinjiang, and have a scattered distribution in the mid-west of Inner Mongolia and Tibet [2]. *P. juncea* is a long- living perennial grass with clustered leaves, multiple tillers and a strong root system [3]. *P. juncea* has been used as a gene donor in disease resistance improvement of wheat germplasm, including resistance to the stripe rust, yellow stunt virus and take-all in wheat [4–7]. It has extremely strong drought resistance and cold tolerance and can also restrain weeds, and has been used to establish long-lasting pasture and rangelands in North America [8, 9]. Thus, *P. juncea* is an excellent perennial grass with economic and breeding value [10].

*P. juncea* is a perennial forage, its reproductive and nutritional growth both occur directly through tillering [11, 12]. Thence, tillering is a significant to the forage and yield of *P. juncea*. Tillering is a special branching characteristic in gramineous forage that independent of the mother stem on the basal node of grass stem [13]. The ability to form tillers is a protective mechanism for plants to avoid injury (such as loss of buds) and to adapt to the environment [14]. Significantly, tillering is one of the main agronomic traits of bunch grasses, which directly affects the yield of forages and seed yield [15, 16]. The most important food crops globally, such as wheat (*Triticum aestivum*), Oats (*Avena sativa* L.) and rice (*Oryza sativa*), are also gramineous, and the grain yield per unit area is closely related to planting density, the rate of reproductive tillers and tiller number per plant [17]. Therefore, genetic mechanisms affecting tillering traits of different Gramineae species have important physiological, ecological and agronomic implications.

Tillering is a very complicated process controlled by a variety of internal factors and external environmental conditions, including natural conditions, endogenous hormones, genetic features and other factors. It has been reported that the growth of tiller buds is closely related to nutrition. When the plant nutrition is sufficient, it can promote the growth of tiller buds, in which nitrogen can regulate the number of tillers [18]. In addition, the growth and development of tiller buds can be inhibited by strigolactones (SLs), auxin (IAA) and abscisic acid (ABA) [19–21], while the growth of tiller buds can be promoted by cytokinins (CTK) [22]. It has been reported that SLs can destroy the polar transport of IAA through cross response with IAA to inhibit the growth of buds [23]. IAA and CTK have antagonistic effects on the growth of lateral buds, and IAA can regulate the synthesis of CTK and affect the growth of lateral buds [24, 25]. Although many studies have revealed that tillering is the result of the balance of several phytohormones instead of a single hormone [26], there is still no suitable model to explain the relationship between various hormones and tillering, because of the complexity of plant hormones that regulate tiller bud growth [27]. The occurrence of tillers may be controlled by multiple genes, and the growth of tiller buds may also be closely related to molecular regulation. These genes are mainly divided into two main groups in rice. One group contains three main tiller formation regulators that have been cloned including the Teosinte Branched 1 (*TB1*) gene-regulating nutritional tiller number, Monoculm 1 *(MOC1)* gene regulating rice tillering buds, and the Tiller Angle Control 1 (*TAC1*) gene. These three are key genes that regulate the formation of tiller buds and promote tiller prolongation in rice through control of the initiation of axillary meristem and induce the formation of tiller buds [28–30]. In order to clarify the molecular mechanism of *MOC1* involved in regulating the rice tillering process, Sun conducted yeast hybrid screening to identify the functional protein of the *MOC1* gene [31]. The results showed that *MOC1* regulated the growth of tillering buds and that the expression of *MIPs* interact with each other. Another group of genes are related to biosynthesis and signal transduction of phytohormones, involving cytokinin oxidase/ dehydrogenase 9 *(CKX9)*, Dwarf 27 *(D27)*, Dwarf 53 *(D53)* and isopentenyl transferase (*IPT*), which control the outgrowth and development of tiller buds [32–35]. Such as, Zhao reported that *D27* gene plays critical role in controlling the tiller number in hexaploid wheat by participating in the biosynthesis of strigolactones [33]. *OsIPT* is a key enzyme regulating cytokinin synthesis in rice. After removing the rice stem tip, *OsIPT* gene expression in rice stems was significantly increased and the outgrowth of rice tiller buds was promoted [36].

In addition, the development of tiller buds is controlled by natural conditions. Light, temperature, plant density, water and fertilizer affect the growth and development of plant tillers. Generally, greater light intensity is more beneficial for the occurrence of tillers [37]. Tillering is also closely related to nutritional levels in plants and the environment. Nitrogen, phosphorus, potassium, boron, zinc and even lead and arsenic are related to the tillers [38]. The tillering process of gramineous plants is a complex biological phenomenon, which involves the precise regulation of many genes and the comprehensive expression of genes, hormones and environmental interactions.

With the development of high-throughput sequencing technology, the transcriptome sequence information of plant genome can be quickly and comprehensively obtained [39]. At present, this technology is widely used in gene expression analysis of biological transcriptomes and to accurately explore important functional genes [40]. At the same time, transcriptome analysis can provide basic information for the genetic characteristics of species without detailed genomes sequence information, particularly in the research of tillering-related genes [41–43]. However, screening and using stable reference genes was helpful to eliminate the variability caused by different development stages, different tissue samples and various stress conditions, so as to ensure the accuracy and reliability of qRT-PCR results. Stable reference genes have been found in many plants, for example orchardgrass (*Dactylis glomerata* L.), rice (*Oryza sativa* L.) and wheat (*Triticum aestivum* L.) [44–46]. There are no reports on the tillering regulation related genes，and any stable reference genes in the qRT-PCR process for *P. juncea.* Therefore, next-generation sequencing technology was used in this research to study the differentially expressed genes (DEG) related to tillering of *P. juncea* to identify the candidate genes that may regulate tillering in perennial grasses, and to study the metabolic pathways corresponding to the regulation of tillering related genes. Furthermore, based on the RNA-seq datasets screened candidate genes of *P. juncea*, the reliability of the transcriptome data was verified by qRT-PCR. The stability of the reference gene for expression verification was comprehensively evaluated using five softwares (BestKeeper, geNorm, Delta Ct, NormFinder and RefFinder) to establish appropriate reference genes for *P. juncea* genome. This study provides basic data for genotype selection, molecular marker-assisted breeding, and breeding efficiency acceleration of *P. juncea* and other perennial grass on biomass yield traits.

## Results

### Phenotypic analysis of the dense and sparse tillering trait of *P. juncea*

**Fig. 1:**
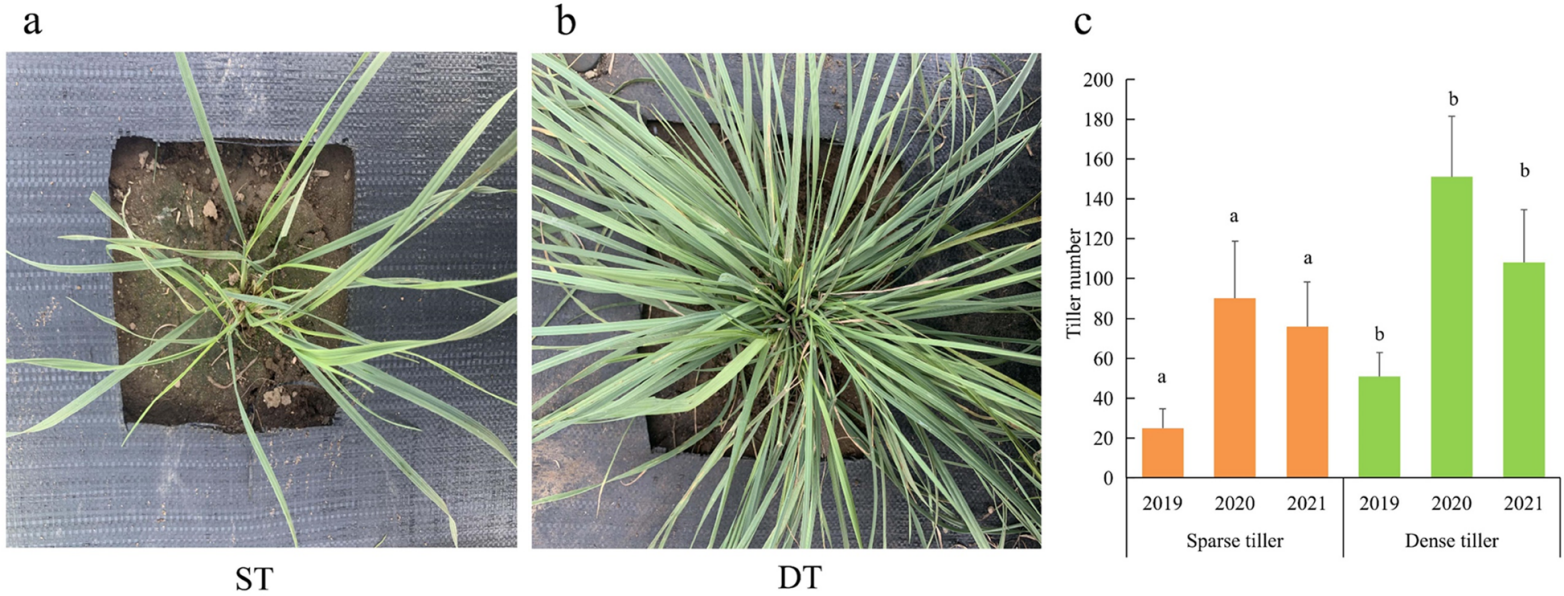
The phenotype photographs of ST and DT. **a** sparse tillering genotype in the experimental field. **b** dense tillering genotype in the experimental field. **c** the tiller number of ST and DT in 3 years (*P*<0.05)

**Fig. 2:**
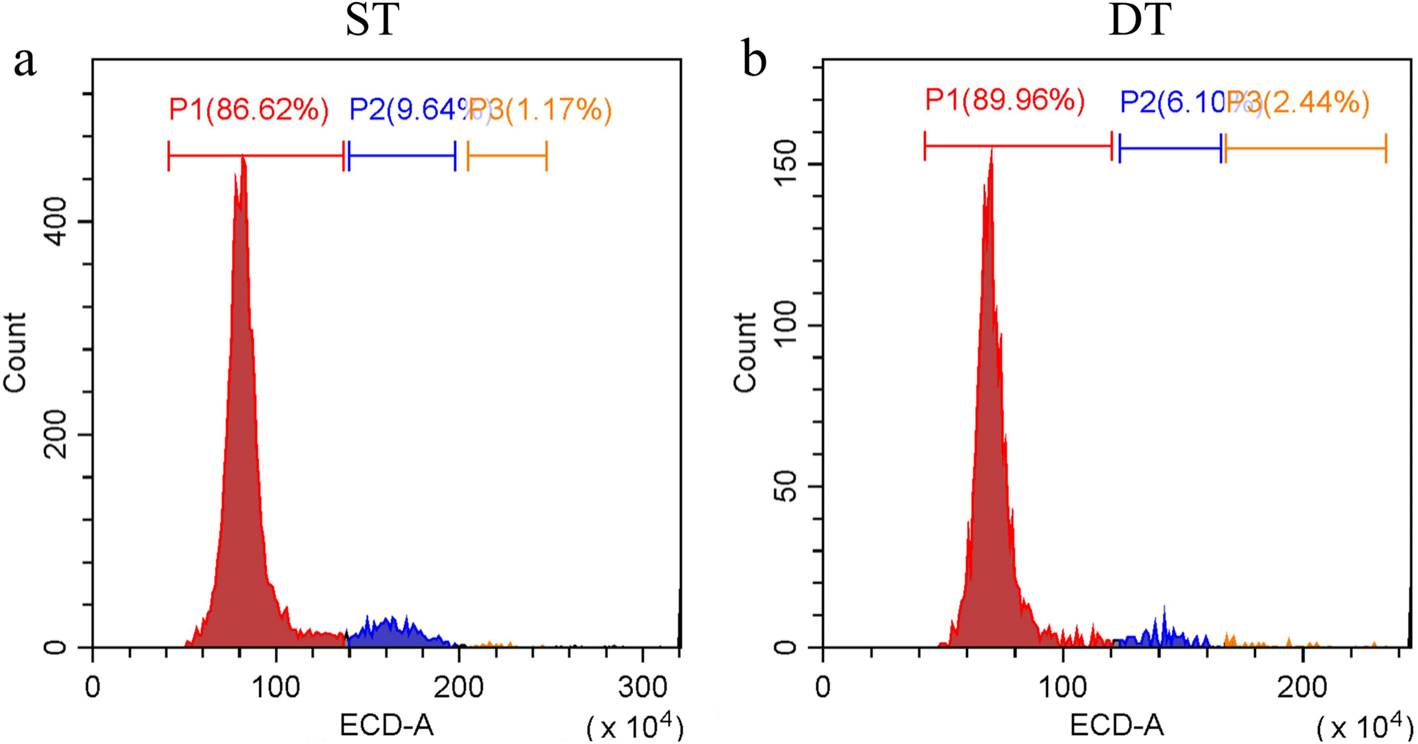
Identification of ploidy test result of *P. juncea* by flow cytometry

Dense and sparse tillering materials formed average 51 and 25 new tillers in 2019, respectively. Therefore, there was a significant difference in the number of tillers between DT and ST (*P* < 0.05) (Fig. 1). The flow cytometry analysis of the two samples showed that both DT and ST were diploid genotypes. The ploidy identification results show that the difference between dense tillering and sparse tillering was not caused by ploidy (Fig. 2). Moreover, the tillering related agronomic traits of the two samples were evaluated in three consecutive years (Table 1). The basal cluster diameter, natural height and absolute height of DT were significantly higher than those of ST. During the measurement, the height of DT was moderate, which is conducive to lodging resistance and high yield. An inverse proportional relationship between tiller number and tiller angle for DT and ST genotypes.

**Table 1:** Mean value of tiller correlation of *P. juncea*

Items	ST			DT		
	2019	2020	2021	2019	2020	2021
Natural height (cm)	20.77 ± 4.49aC	42.69 ± 15.71bB	102.63 ± 21.42bA	24.27 ± 11.09aC	53.25 ± 11.99aB	111.32 ± 19.28aA
Absolute height (cm)	24.27 ± 6.13aC	53.25 ± 14.59bB	111.32 ± 22.60bA	30.25 ± 3.56aC	68.01 ± 12.05aB	128.25 ± 22.4aA
Basal cluster diameter (cm)	14.43 ± 3.85bC	37.56 ± 9.16bB	60.21 ± 13.51bA	18.84 ± 2.97aC	48.21 ± 18.52aB	70.91 ± 12.95aA
Nutritional tiller number	25 ± 9.70bC	90 ± 28.76bB	76 ± 22.27bA	51 ± 11.94aC	151 ± 30.52aB	108 ± 26.53aA
Tiller angle (°)	41 ± 8.55aC	52 ± 7.35aB	62 ± 8.65aA	37 ± 8.15aC	50 ± 9.71aB	59 ± 12.01aA
Leaf length (cm)	21.68 ± 3.12aB	31.93 ± 3.93aA	32.90 ± 5.97aA	21.49 ± 3.41aB	33.07 ± 3.79aA	32.22 ± 5.64aA
Leaf width (cm)	0.44 ± 0.05aA	0.41 ± 0.05aB	0.41 ± 0.04aB	0.42 ± 0.05aA	0.42 ± 0.04aB	0.42 ± 0.05aB
Canopy width (cm)	37.86 ± 14.20aC	59.36 ± 11.15aB	61.00 ± 14.76aA	36.99 ± 13.72aC	61.40 ± 11.69aB	78.70 ± 12.73aA

Different capital letters indicate the significant difference of the same index in different years, and different lowercase words w indicate the significant difference of the same index in the same year (*P* < 0.05)

### De novo assembling of the *P. juncea* transcriptome

In our study, based on sequencing by synthesis (SBS) technology, the Illumina Hiseq high-throughput sequencing platform was used to sequence the cDNA library, which produced a large variety of high-quality reads, and the transcriptome sequencing of six *P. juncea* samples was completed. After a series of quality controls, including truncating sequencing adapters in reads and filtering low-quality data, an average of 21,643,495 high-quality clean reads were obtained per sample. The clean bases of each sample had an average total number of 6,468,655,828, and the percentage of Q30 bases in each sample was not less than 91.98%, respectively (Additional file 1).

A total of 100,560 Unigenes were obtained by de novo assembled from these clean reads, and the N50 of unigene was 1739 bp, which had high assembly integrity (Table 2). By drawing the saturation curves, it was found that the capacity and the mapped reads of six libraries tend to be saturated, and that the amount of effective sequencing data was sufficient (Additional file 2). These results indicate that the amount of effective sequencing data of transcriptome sequencing library was sufficient and of high quality. In addition, the repeatability and FPKM values of all samples were evaluated using Pearson correlation coefficient (*R*^*2*^) and principal component analysis (PCA), and results suggesting that there were significant differences in the expressed of genes between DT and ST genotypes (Additional file 3). In general, the quality of the sequencing data was reliable and could be used for subsequent DEGs analysis.

**Table 2:** Sequential assembly results

Length Range (bp)	All Unigenes	DT Unigenes	ST Unigenes
200–1000	70,671(70.28%)	113,384(83.40%)	110,823(83.64%)
1000–2000	16,936(16.84%)	14,270(10.50%)	13,762(10.39%)
2000+	12,953(12.88%)	8294(6.10%)	7923(5.98%)
Total number	100,560	135,948	132,508
Total length	91,562,881	87,683,094	84,830,605
N50 length	1739	1044	1022
Mean length	910.53	644.98	640.19

### Gene annotation of the *P. juncea* transcriptome

**Fig. 3:**
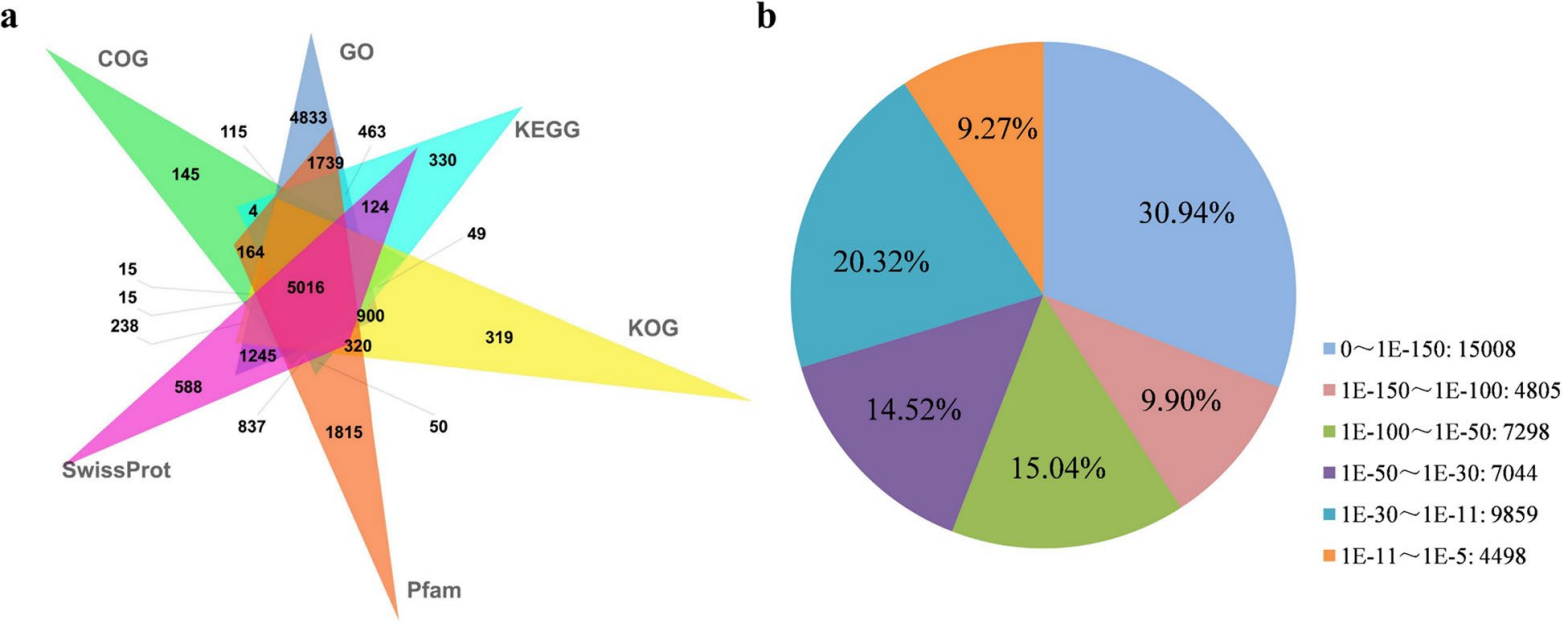
Homology search of *P. juncea* unigenes. **a** Venn diagram of the unigenes annotated in public databases by BLAST. The numbers in the graph represent the number of unigenes annotated by single or multiple databases. **b** E-value distribution of the Nr database

A total of 100,560 unigenes were identified, of which 49,363 unigenes were annotated in at least one database using BLASTx (E-value <1 × 10^−5^) and HMMER (E-value < 1 × 10^−10^) (Table 3). In addition, 145, 588, 1815, 319, 330 and 4833 unigenes were annotated to the COG, SwissProt, Pfam, KOG, KEGG and Go databases (Fig. 3a). About 48.24% (48,512/100,560) of the unigenes could be annotated by BLASTx (E-value < 1E-5) using the NCBI Nr database. Based on the E-value distribution, approximately 29.59% of unigenes showed homology (1E-30 < E-value ≤1E-5), 29.56% of unigenes showed strong homology (1E-100 < E-value ≤1E-30), and 40.84% of the unigenes showed extremely strong homology (E-value < 1E-100) to available plant genome sequences (Fig. 3b).

**Table 3:** BLAST analysis of the unigenes annotation in public databases

Annotated database	Number of Unigene	Annotation percentage(%)	300 ≤ length<1000	length ≥ 1000
COG annotation	12,314	24.94	1738	8792
GO annotation	34,288	69.46	8422	19,058
KEGG annotation	14,514	29.40	3362	8755
KOG annotation	21,747	44.06	4383	13,409
Pfam annotation	27,966	56.65	5477	18,673
SwissProt annotation	26,617	53.92	6225	17,013
eggNOG annotation	42,125	85.34	10,558	23,060
Nr annotation	48,512	98.28	13,124	24,787
All annotated	49,363	100	13,319	24,892

### Functional characterization of DEGs among the DT and ST genotypes

**Fig. 4:**
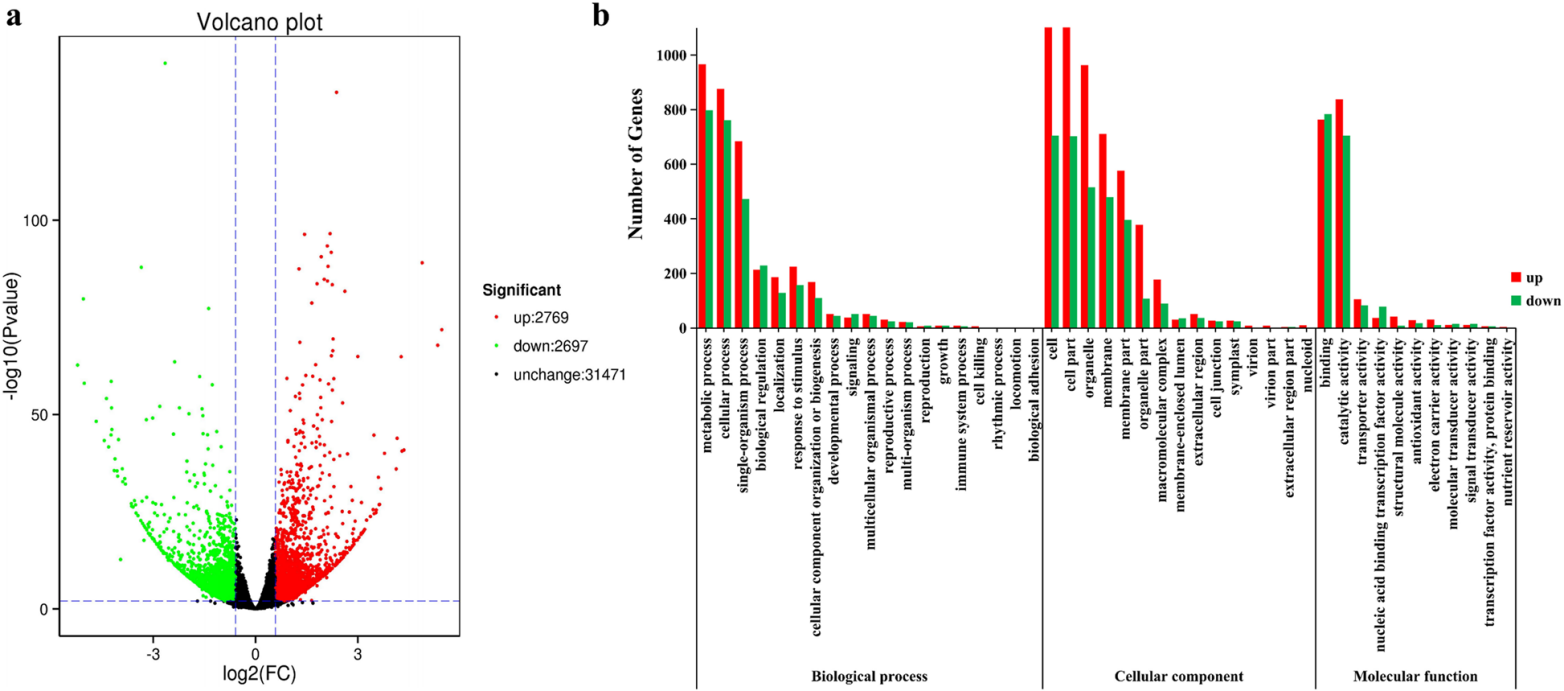
DEGs analysis of *P. juncea*. **a** DEGs of DT and ST in Volcano map. **b** GO analysis of DEGs specifically expressed between DT and ST

**Fig. 5:**
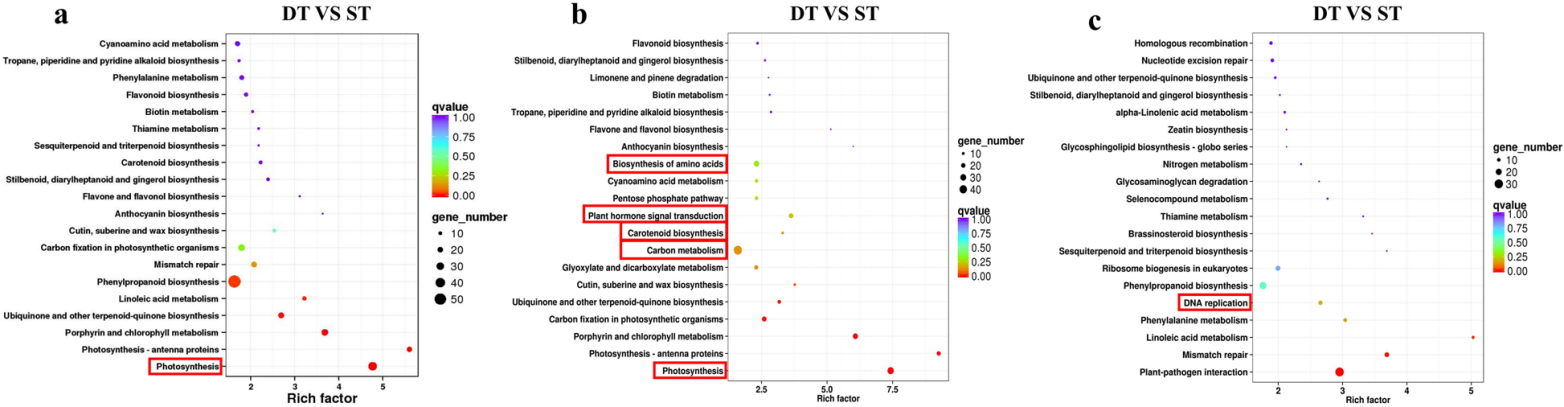
Scatter plot of KEGG functional classification of DEGs. Including (**a**), all annotation. **b** up annotation (**c**), down annotation. The larger the rich factor, the more significant the enrichment level of DEGs in the pathway; The color of the dot represents the q value, and the smaller the q value, the more reliable the enrichment significance of DEGs in the pathway; the greater dot represents the more DEGs

In order to analyze the genetic differences in the process of tillering formation, the DEGs of tiller bud tissues between DT and ST genotypes were compared. DEGs have higher expression level in DT compared to ST, indicating that the genes were up-regulated; Otherwise, the genes were down-regulated. A total of 5466 significant DEGs were assigned to one or more functions, 2769 unigenes were upregulated, and the other 2697 unigenes were downregulated (Fig. 4a). In order to investigate the functions of DEGs between DT and ST at the same tillering developmental stage, GO and KEGG pathway were analyzed in detail (Fig. 4b). In the cellular component function, the DEGs were significantly enriched in “cell” (1142 upregulated, 704 downregulated unigenes) and “cell part” (1139 upregulated, 702 downregulated unigenes). In the molecular function, the DEGs were significantly associated with the “binding” subcategories (764 upregulated, 783 downregulated unigenes) and “catalytic activity” (838 upregulated, 705 downregulated unigenes). In the biological process function, “metabolic process” and “cellular process” were the most common, which were related to 1765 and 1637 DEGs (Additional file 4). Otherwise “rhythmic process” (1 unigenes) and “biological adhesion” (1 unigenes) were infrequently related. Moreover, we conducted enrichment analysis based on the KEGG database to explore the biological functions of DEGs. In the comparison of DT and ST, a total of 7885 unigenes, including 723 DEGs, were assigned to 119 KEGG pathways (Additional file 5). KEGG analysis revealed that “Photosynthesis” and “Carbon metabolism” were significantly enriched in the DT vs ST comparison (Fig. 5a, b). And compared with ST, 33 of the 35 DEGs related to photosynthesis were upregulated in the DT, 44 of 56 DEGs related to carbon metabolism were upregulated in the DT. Compared with ST, “Carotenoid biosynthesis” was enriched in the DT genotype (Fig. 5b). But DEGs related to “DNA replication” was significant enriched in the DT and ST downregulated comparison. Compared with ST, twelve of the seventeen DEGs related to DNA replication were downregulated in the DT (Fig. 5c). The DEGs associated with “Photosynthesis”, “Carbon metabolism” and “DNA replication” were listed in the Additional file 6.

Generally speaking, the comprehensive analysis of the KEGG pathway and GO functional enrichment showed that there were DEGs related to Carbon metabolism, Photosynthesis and DNA replication in eukaryotes between DT and ST. Besides, in DT vs ST upregulate comparison, metabolic processes of “Biosynthesis of amino acids” and “Plant hormone signal transduction” were also have relatively high q value and suggests that these processes were involved in tillering regulation of *P. juncea* (Fig. 5b).

### Weighted gene co-expression network analysis of two genotypes

**Fig. 6:**
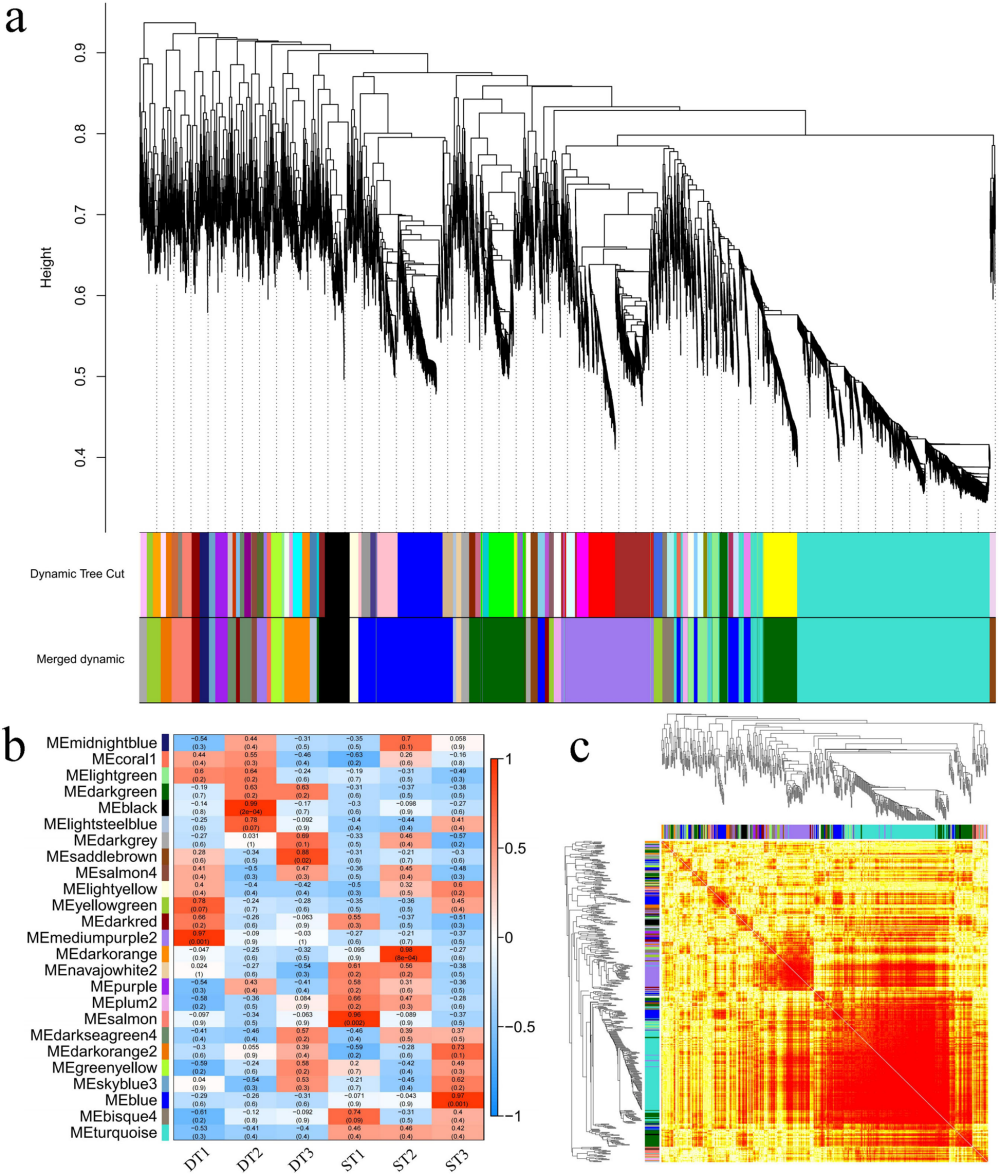
WGCNA of genes at six samples. **a** Gene co-network expression module generated by gene systematic clustering tree and dynamic cutting method, which are marked with different colors, and finally divided into 25 modules. **b** Heatmap of correlations between modules and samples. The x-axis represents the six samples, and the y-axis represents the cluster module. The closer the correlation between the sample and the module is to the absolute value of one, that is, the deeper the red, the higher the correlation between the module and the sample. **c** The heatmap of gene co-expression network, in which the left and upper sides are the results of symmetrical systematic clustering trees and modules. The lower right heatmap area shows the dissimilarity between genes, the smaller the value, the deeper the color

**Fig. 7:**
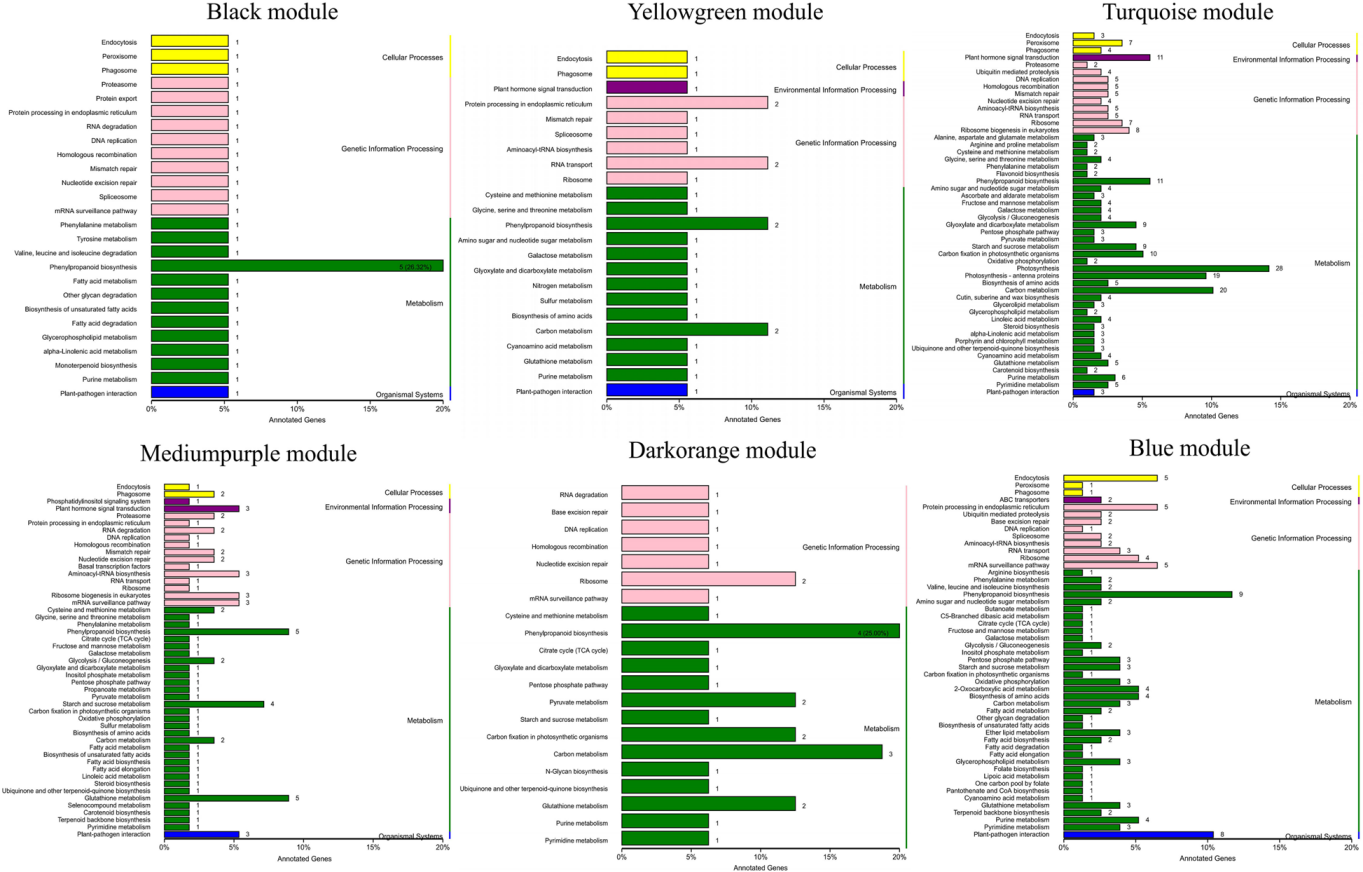
KEGG functional classification of DEGs in different modules of WGCNA. The y-axis represents the KEGG metabolic pathway, and the x-axis is the number of genes annotated to this pathway

WGCNA was used to explore the biological correlation between co-expression module and target traits. In this study, WGCNA R package was used to construct a co-expression network for 7935 genes of all samples. Among the genes shown in Fig. 6a, different colors represent each specific module, each of which contains a cluster of highly correlated genes. Through WGCNA, 25 co-expression modules were constructed, of which the turquoise module was the largest module, consisting of 2042 unigenes, whereas the coral 1 module was the smallest, consisting of only 39 unigenes (Additional file 7). This analysis showed eight different modules (mediumpurple 2, saddlebrown, darkorange, salmon, black, turquoise, yellowgreen and blue modules) with strong correlation values. Especially, the turquoise module was only highly correlated within ST (Fig. 6b). In the network heatmap, turquoise modules were clustered into a large group, and the darker color indicates dissimilarity value between genes was small (Fig. 6c). Notably, six of the eight distinct modules (black, mediumpurple 2, darkorange, turquoise, yellowgreen and blue modules) were involved in phenylpropanoid biosynthesis. Furthermore, the turquoise module is involved in photosynthesis and plant hormone signal transduction, which suggests that DT and ST may have differences in hormone biosynthesis and photosynthetic efficiency (Fig. 7).

### KEGG pathway enrichment analysis of DEGs with tillering

**Fig. 8:**
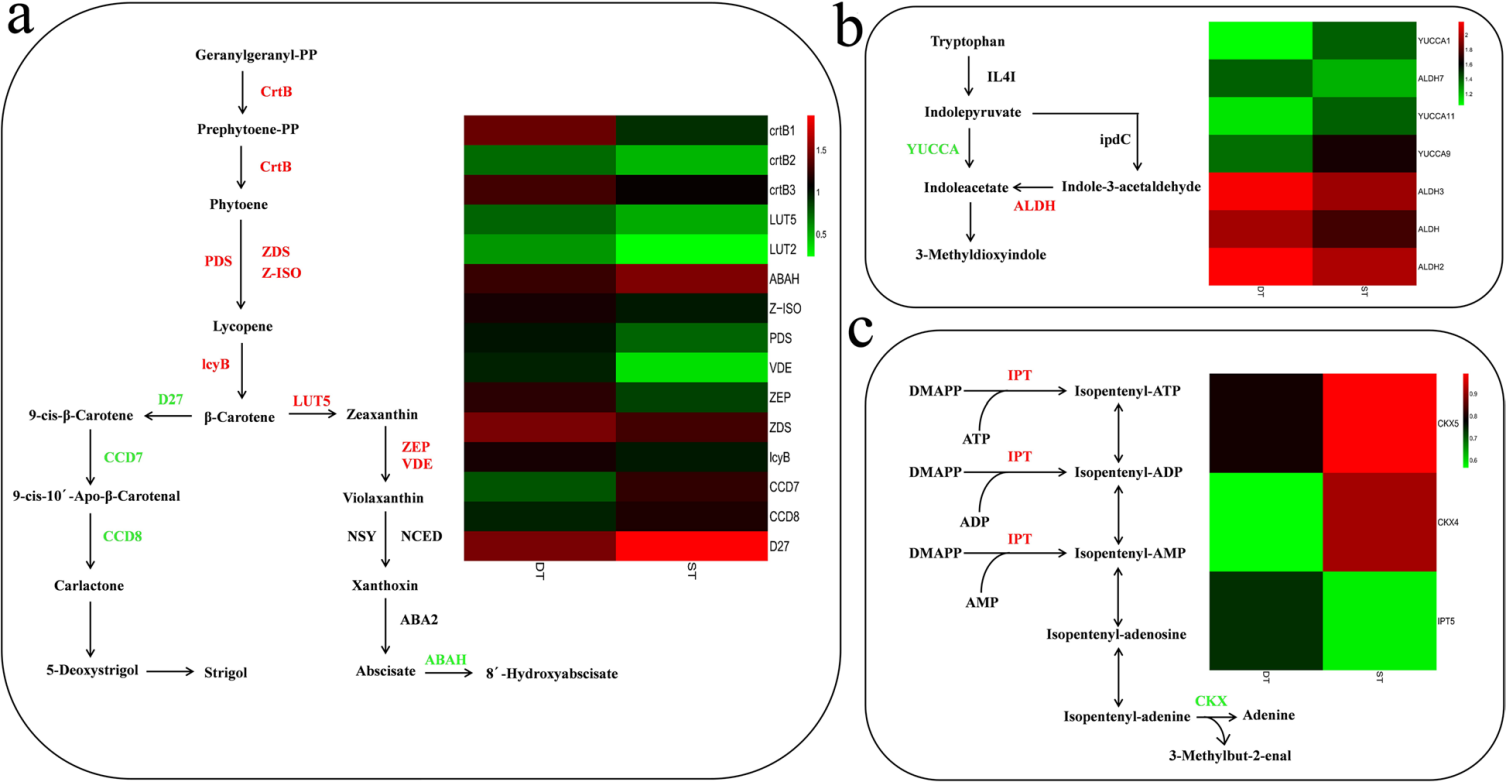
Phytohormone pathway in DT and ST. **a** SL biosynthesis pathway. **b** 3-Methyldioxyindole biosynthesis. **c** Cytokinin biosynthesis. Different columns in the figure represent two genotypes, and different rows represent different genes. The color represents the expression level of gene in the sample, and the logarithm of FPKM based on 2

In order to further insight into the effect of DEGs on tillering regulation, we performed an enrichment analysis of DEGs based on the KEGG database. SLs and auxin have been reported to inhibit tiller shoot formation, while cytokinin can promote tiller formation. Thus, we first compared SL biosynthesis pathway (Fig. 8a). First, geranylgeranyl pyrophosphate (GGPP) is converted into Phytoene through the action of 15-cis-phytoene synthase (crtB). Then, phytoene is converted into lycopene through the action of 15-cis-phytoene desaturase (PDS), zeta-carotene desaturase (ZDS) and zeta-carotene isomerase (Z-ISO), and last, lycopene is converted into β-carotene through the action of lycopene beta-cyclase (lcyB). Overall, the DEGs encoding these five enzymes were up-regulated in DT. Second, β-carotene was transformed into SL via four steps involving three enzymes (beta-carotene isomerase (*DWARF27*), 9-cis-beta-carotene 9′,10′-cleaving dioxygenase (*CCD7*), carotenoid cleavage dioxygenase 8 (*CCD8*)), and β-carotene was transformed into 8′-Hydroxyabscisate via four steps including seven enzymes (beta-ring hydroxylase (LUT5), zeaxanthin epoxidase (ZEP), violaxanthin de-epoxidase (VDE), neoxanthin synthase (NSY), 9-cis-epoxycarotenoid dioxygenase (NCED), xanthoxin dehydrogenase (ABA2) and (+)-abscisic acid 8′-hydroxylase (ABAH)). In the ABA biosynthesis pathway, ABAH expression was down-regulated in DT. Second, we compared 3-Methyldioxyindole biosynthesis in the tryptophan metabolism pathway (Fig. 8b). First, tryptophan is transformed into indolepyruvate through catalyzation by L-amino-acid oxidase (IL4I). Then, indolepyruvate generates indoleacetate through catalysis by indole-3-pyruvate monooxygenase (*YUCCA*) or indolepyruvate decarboxylase (ipdC) and aldehyde dehydrogenase (NAD+) (ALDH). Finally, indolepyruvate is transformed into 3-Methyldioxyindole. Overall, 1 DEG encoding enzymes *YUCCA* was downregulated, and 1 DEG encoding enzyme ALDH was upregulated. Accordingly, the auxin synthesis of ST increased significantly compared with DT, which was mainly owing to the 1 DEG encoding *YUCCA*. It has been reported recently that cytokinin can relieve the apical dominance caused by IAA and affect the growth and the number of tillers by promoting the germination and elongation of tiller buds [47]. Among these, *IPT* (isopentenyl transferase) and *CKX* (cytokinin dehydrogenase) are key enzymes regulating cytokinin synthesis. Zeatin riboside (ZR) and Zeatin are cytokinin substances. Higher ZR can promote the germination of tillering buds. Therefore, the third pathway we compared was cytokinin biosynthesis in the zeatin biosynthesis pathway (Fig. 8c). The isopentenyl of DMAPP (dimethylallyl diphosphate) is transformed to the adenosine part by *IPT*, and iPTP (isopentenyl adenosine-5 ‘- triphosphate), iPDP (isopentenyl adenosine-5’ - diphosphate) or iPMP (isopentenyl adenosine-5 ‘- monophosphate) are synthesized with plant ATP, ADP or AMP respectively. Then, isopentenyl-AMP generates adenine through catalysis by *CKX*. Overall, 1 DEG encoding enzymes *CKX* was downregulated, and 1 DEG encoding enzyme *IPT* was upregulated.

**Fig. 9:**
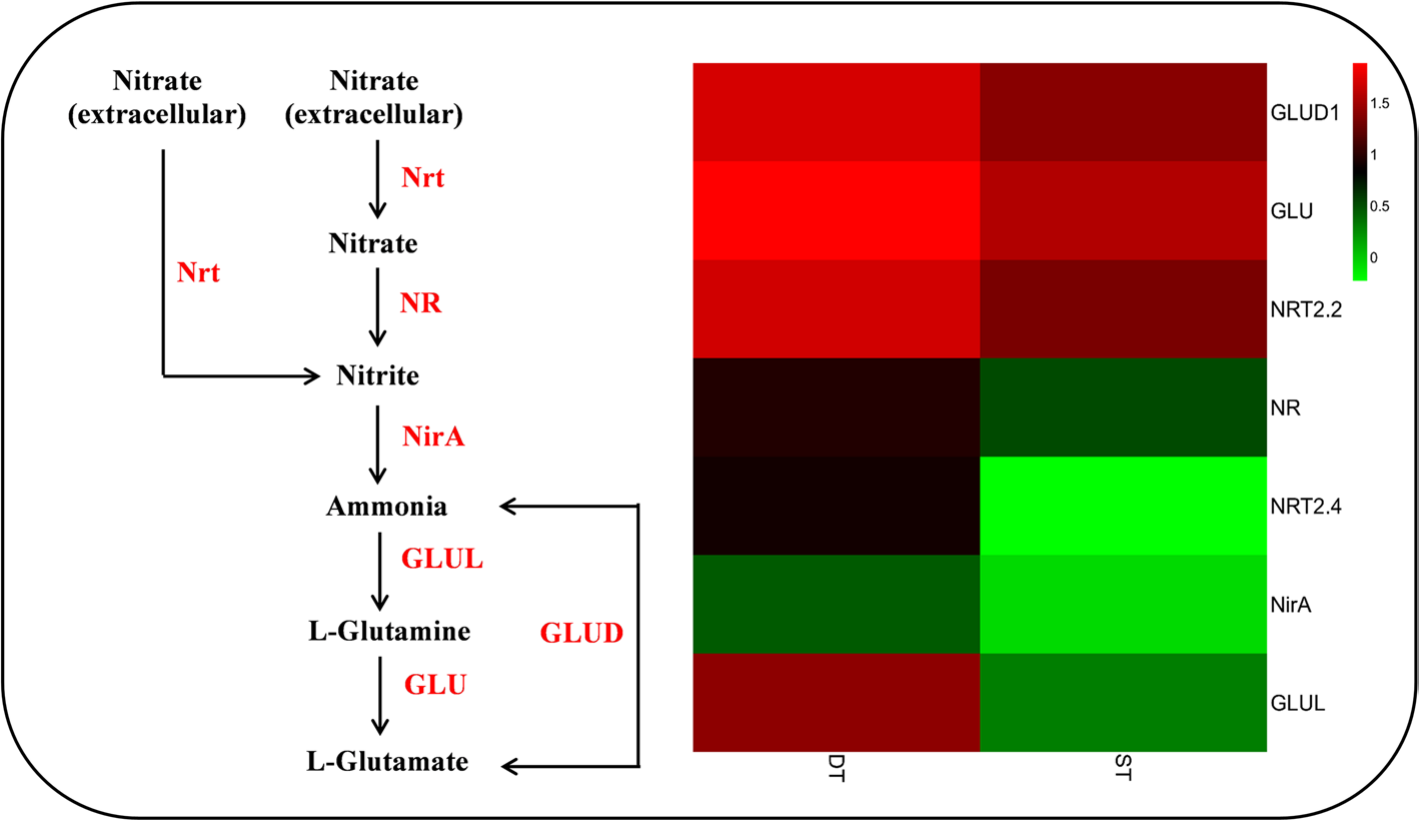
Nitrogen metabolism pathway in DT and ST. Different columns in the figure represent two genotypes, and different rows represent different genes. The color represents the expression level of gene in the sample, and the logarithm of FPKM based on 2

Nitrogen significantly affected the occurrence of plant tillers [18]. The fourth compared nitrogen metabolism pathway (Fig. 9). Nitrate or nitrite is converted to nitrite through the nitrate reductase (Nr) and nitrate/nitrite transporter (Nrt). Then, nitrite is transformed into ammonia by ferredoxin-nitrite reductase (NirA). Ammonia is transformed into L-glutamine through glutamine synthetase (GLUL), which is catalyzed to form L-glutamate by glutamate synthase (GLU). Ammonia is converted into L-glutamate by glutamate dehydrogenase (NAD(P)+) (GLUD). Nrt, Nr, GLU, and GLUD were upregulated in tiller buds of DT relative to ST, indicating that DT could have a higher nitrogen utilization rate.

**Fig. 10:**
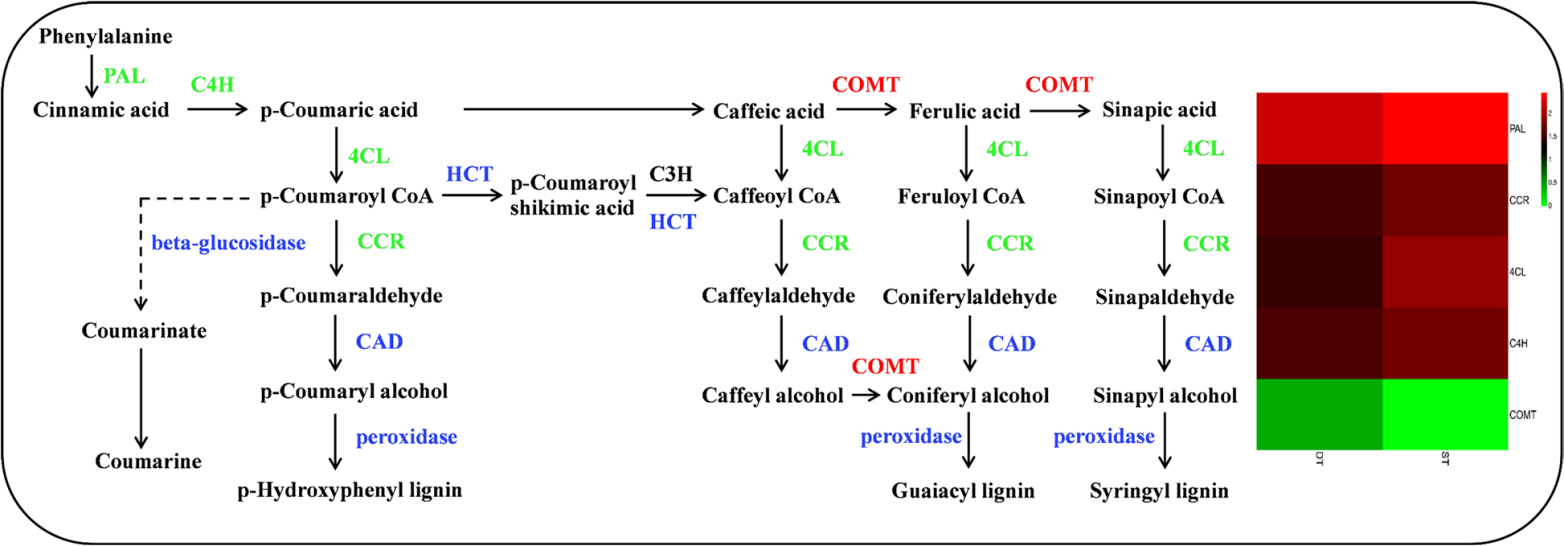
lignin biosynthesis pathway in DT and ST. Different columns in the figure represent two genotypes, and different rows represent different genes. The color represents the expression level of gene in the sample, and the logarithm of FPKM based on 2

Lignin is particularly important in the formation of cell wall and is involved in several physiological processes [48]. And the number of tillers of gramineous plants is related to the content of lignin. The fifth pathway we compared was lignin biosynthesis in the phenylpropanoid biosynthesis pathway (Fig. 10). First, phenylalanine is transformed into cinnamic acid through catalyzation by phenylalanine ammonia-lyase (*PAL*). Cinnamic acid is transformed into p-Coumaric acid through catalyzation by trans-cinnamate 4-monooxygenase (C4H). p-Coumaric acid is converted into p-Hydroxyphenyl lignin through the action of four enzymes: 4-coumarate--CoA ligase (*4CL*), cinnamoyl-CoA reductase (*CCR*), cinnamyl-alcohol dehydrogenase (CAD) and peroxidase. Second, p-Coumaroyl CoA is transformed into guaiacyl lignin through six steps involving six enzymes: shikimate O-hydroxycinnamoyltransferase (HCT), 5-O-(4-coumaroyl)-D-quinate 3′-monooxygenase (C3’H), *CCR*, CAD, caffeic acid 3-O-methyltransferase (COMT) and peroxidase. At the same time, ferulic acid can also be transformed into guaiacyl lignin through catalyzation by *4CL, CCR*, CAD and peroxidase. Ferulic acid is transformed into sinapic acid through catalyzation by COMT and transformed into syringyl lignin via four steps. Overall, 4 DEGs encoding enzymes *PAL*, C4H, *4CL* and *CCR* were downregulated in DT and COMT was upregulated in DT. In summary, these results indicated that there might be a lower accumulation of lignin content in DT relative to ST.

### Stability analysis of different reference genes

We selected 12 protein-coding genes in *P. juncea* with the highest expression levels as candidate reference genes (Additional file 8). To precisely examine candidate reference gene stabilities, four algorithms (geNorm, NormFinder, Bestkeeper and Delta CT) and the RefFinder technique were applied in the analysis, which ranked the stability of genes. The determination of the best reference gene mainly depends on the average gene expression stability value (M value) calculated by the geNorm program. The smaller the M value, the better the stability of the reference gene. The top three stable candidate reference genes in all tested samples by geNorm were *Actin97, GAPDH* and *EF-1α* (Additional file 9: Fig. S3a), whereas the two least stable genes were *UBI* and *UBC2*. The NormFinder program works similarly to geNorm. Results from the Normfinder program showed that the *Actin1* and *αTUB* genes were the most stable reference genes, whereas the two least stable genes were *UBI* and *UBC2* (Fig. S3b)*.* Bestkeeper recommends reference genes expressing stably on the basis of SD and CV values*.* The top five stable candidate reference genes in all tested samples by Bestkeeper were *18S rRNA*, *GAPDH*, *Actin97*, *βTUB* and *EF-1α* (Fig. S3c). All of the twelve reference genes in Table S3 (Additional file 10) had *P* values less than 0.05, and the *αTUB* reference genes had the highest *r* value, indicating the highest stability. The Delta-Ct method screens reference genes based on calculating the difference in △Ct between all samples, and calculates the average standard deviation of each reference gene, such that the smaller the average standard deviation, the higher the stability of the reference gene. Fig. S3d showed the mean standard deviation of the 12 candidate reference genes, and *Actin1* had the smallest values and the best stability. Reffinder is a comprehensive application based on screen reference genes, and it integrates and comprehensively analyzes four other software for screening reference genes, so as to compare and rank candidate reference genes. RefFinder recommended the gene expressional stability rankings in the order *Actin1* > *GAPDH* > *Actin97* > *EF-1α* > *αTUB* > *βTUB* > *18S rRNA* > *UBC17* > *CYP* > *UBC28* > *UBI* > *UBC2.* However, no matter which program was used, the least stable genes were *UBI* and *UBC2* (Table 4).

**Table 4:** Comprehensive ranking of reference genes using RefFinder

Ranking	Delta CT	BestKeeper	NormFinder	GeNorm	Comprehensive ranking
1	*Actin1*	*18S rRNA*	*Actin1*	*Actin97/GAPDH*	*Actin1* (2.30)
2	*αTUB*	*GAPDH*	*αTUB*		*GAPDH* (2.74)
3	*EF-1α*	*Actin97*	*CYP*	*EF-1α*	*Actin97* (3.83)
4	*GAPDH*	*βTUB*	*βTUB*	*Actin1*	*EF-1α* (3.87)
5	*βTUB*	*EF-1α*	*EF-1α*	*UBC17*	*αTUB* (3.94)
6	*UBC17*	*UBC17*	*UBC28*	*αTUB*	*βTUB* (4.86)
7	*CYP*	*Actin1*	*GAPDH*	*βTUB*	*18S rRNA* (5.18)
8	*Actin97*	*UBC28*	*UBC17*	*18S rRNA*	*UBC17* (6.16)
9	*18S rRNA*	*CYP*	*Actin97*	*CYP*	*CYP* (6.42)
10	*UBC28*	*αTUB*	*18S rRNA*	*UBC28*	*UBC28* (8.32)
11	*UBI*	*UBI*	*UBI*	*UBI*	*UBI* (11.00)
12	*UBC2*	*UBC2*	*UBC2*	*UBC2*	*UBC2* (12.00)

### RNA-seq expression validation of DEGs by quantitative real-time PCR analysis

**Fig. 11:**
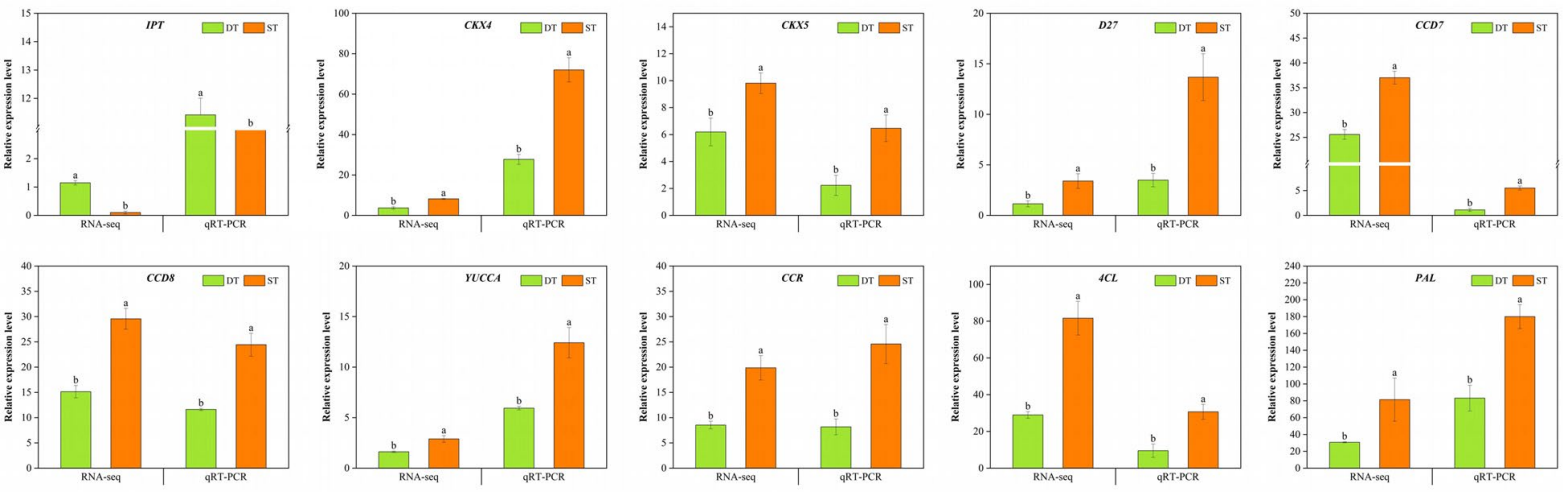
Expression of tillering-related unigenes of *P. juncea* quantified by RNA-Seq and qRT-PCR analysis

The results of KEGG pathway analysis showed that the difference of two tillering phenotypes of *P. juncea* was closely related to the plant hormones biosynthesis. Furthermore, WGCNA indicated that the lignin biosynthesis was also an important factor for the tillering difference. In hormones regulating processes, SL and IAA both inhibits the growth of tillering buds. Therefore, genes involved in plant hormones biosynthesis process were mostly down-regulated in dense tillering genotype. In order to quantitatively determine the accuracy of transcriptome data and verify the results of tillering molecular mechanism, we selected ten genes (*IPT, CKX4, CKX5, D27,CCD7, CCD8, YUCCA, CCR, 4CL* and *PAL*) involved in the four pathways discussed in the previous section for further verification. *Actin1* was used as the housekeeping gene (Additional file 11). RNA samples extracted from tiller nodes of DT and ST samples of *P. juncea* were used as templates, first-strand cDNA was synthesized from the total RNA by the manufacturer’s instruction (Cowin Bio, China), and selected genes related to tillers in the DT and ST samples were validated based on qRT-PCR. Only *IPT* had higher expression levels in DT than ST among the candidate DEGs, while the other nine genes all had lower expression levels in DT (Fig. 11). The significant difference in qRT-PCR data between DT and ST of *P. juncea* was analyzed by t-test. The expression levels of all genes detected by qRT-PCR are highly consistent with transcriptome data, which proved the accuracy and effectivity of the transcriptome data.

## Discussion

### Phenotypic differences between dense and sparse tillering *P. juncea*

Tillering is a significant agronomic trait that determines forage yield and seed yield of gramineous forages, which is the result of the combined effects of heredity, plant hormones and external environmental factors. Tillering development includes three steps: axillary meristem initiation, the formation of tiller bud and the elongation of tiller bud [49]. The process exerts a great impact on aboveground biomass, plant morphology and seed yield, all of which are important agronomic traits. In this research, comparing DT and ST we found that plant height has a proportional relationship to tiller number (Table 1). The KEGG enrichment analysis of DEGs also found that a large number of DEGs were enriched in the photosynthesis pathway, and the expression in DT was significantly higher than that in ST. Thus, high photosynthetic efficiency may be the reason for the more vigorous growth of DT. We found in this research that all materials with significant differences in tillering ability between DT and ST have same ploidy level (Fig. 2). Xu et al. [44] reported that ploidy was not associated with a high-tillering and a low-tillering phenotype of Orchardgrass. Abundant researches have shown that the tiller number is related to the tiller angle [50, 51]. To improve the forage and seed yield of gramineous species, increasing plant density is an effective approach, but an excessively large tillering angle will reduce photosynthetic efficiency and increase leaf shade. Therefore, a relatively narrow tiller angle will increase plant yield. Yu et al. [50] reported that in the regulation mechanism of plant tillering branches, the tillering angle is regulated by a major quantitative trait of *TAC1* (Tiller Angle Control 1) gene. The expression or deletion of *TAC1* will lead to change in tiller numbers and tiller angle. Jin et al. [51] found that the *PROG1* (Promote Growth 1) gene regulated tiller angle and tiller number in wild rice, and encoded a zinc transcription factor with transcriptional activity. In this research, we found an inverse proportional relationship between tiller number and tiller angle for DT and ST genotypes, which is consistent with previous reports, and as the number of tillers increases, the forage yield also increases.

### Candidate genes and regulatory processes involved in tiller traits of *P. juncea*

In our research, approximately 5466 DEGs were identified from tiller node tissues of the dense tillering and sparse tillering through a detailed screening. In transcriptome sequencing analysis, we used the tissues of tiller nodes as materials and a great number of genes took part in tiller number regulation were detected, which suggested that tiller node tissue is not only the locus of tiller morphogenesis, but also an extremely important tissue participated in regulating tiller formation. In addition, many of these DEGs were highly expressed in the tiller node tissues of DT compared with ST, which might be responsible for the difference in tiller phenotypes. Xu et al. [44] analyzed transcriptome of different organs of orchardgrass, including tiller buds, shoot base, root and leaf tissues, and found that tiller buds had the most DEGs compared with other organs. Moreover, tiller buds had more tiller regulation pathways (such as hormone regulation and biosynthesis of lignin) than other organs. In this research, only tiller nodes were selected for differential expression analysis with the aim of simplifying the analysis process and better focusing on the DEGs that regulate tiller formation.

KEGG pathway enrichment analysis of DEGs indicated that “DNA replication”, “Carbon metabolism” and “Photosynthesis” were significantly enriched in DT compared to ST (Fig. 5a, b, c). “Photosynthesis” was enriched in the DT and ST upregulated comparison (Fig. 5b). Thirty-three of the 35 DEGs associate with photosynthesis were upregulated in the DT and ST comparison (Additional file 5). On the one hand, the greater average number of tillers, the taller the plants and the greater the biomass of the DT phenotype, which may be related to its higher photosynthetic efficiency. High photosynthetic efficiency implies faster accumulation of organic compounds, which is more conducive to the formation of new organs. On the other hand, the analysis of KEGG pathway enrichment showed that the plants of DT genotype strong growth, which may be due to the important role of DNA replication and photosynthesis in DT genotype. Therefore, active DNA replication and photosynthesis are responsible for the difference of tiller number between the two genotypes. Besides, a number of DEGs are participated in plant hormone synthesis, include “Plant hormone signal transduction” and “Carotenoid biosynthesis” were enriched in the DT and ST upregulated comparison (Fig. 5b). Through WGCNA analysis, some modules closely related to photosynthesis, phenylpropanoid biosynthesis and plant hormone biosynthesis were found. Therefore, combining the results of WGCNA and KEGG pathways, we prove that differences of the genes expression pattern related to SLs, IAA, CTK biosynthesis distinguished the DT genotype of high tillering from the ST genotype of low tillering during the tillering stage, given that SLs, IAA and CTK play important roles in the formation of tillers [27]. Thus, our results showed that the reason for the different tillering phenotypes of *P. juncea* is the differential expression of genes related to the biosynthesis of three hormones between dense tillering and sparse tillering genotypes.

Recently, through in-depth research on physiology, biochemistry and genomics of multi-branch mutants of various model plants and crops, some important genes regulating branch development have been cloned [52–54]. These genes have been shown to be involved in the synthesis pathway of SLs. At present, the reported SLs biosynthesis genes include Arabidopsis multi axillary bud branches *MAX3* (*More axial branching 3*) [55] and rice (*Oryza sativa*) dwarf multi tiller *HTD1 / D17* (*High tillering dwarf 1*) [56]. They encode homologous carotenoid cleaving dioxygenase 7 (*CCD7*), *D10* [57] and homologous carotenoid cleaving dioxygenase 8 (*CCD8*). A large quantity of previous researches have revealed that SL is a new kind of plant hormone, which is mainly synthesized in roots and transported from bottom to top to inhibit the growth of tillering buds [58, 59]. In this research, it was found that three DEGs (*D27, CCD7*, and *CCD8*) of DT genotype involved in SL biosynthesis were downregulated, indicating decreased in SLs biosynthesis. Thus, we infer that the increased of tiller number in the DT might be the result of the decreased of SL biosynthesis.

Studies have shown that auxin (IAA) is mainly synthesized in plant stem tips and young leaves, which is actively transported from top to bottom, and inhibits the growth and development of tillering buds through apical dominance. After the plant apical tissue is removed, the inhibitory effect of apical dominance on lateral buds is relieved, and the plant begins to grow and develop normally, eventually developing into new tillers [60]. In this research, the low expression of IAA synthesis genes in DT may lead to reduced accumulation IAA compared with ST. This suggests that DT buds grow faster than ST buds because the weakening of auxin promotes the growth of lateral shoots. Although it has been reported that IAA levels are generally reduced under decreased *YUCCA* activity [61], we observed in DT that the decrease of IAA biosynthesis was related not only to the downregulation of *YUCCA,* but also to the upregulation of *ALDH*. This reflects that IAA accumulation in different species may be controlled by different key genes. The results suggested that *YUCCA* and *ALDH* are two key genes in the IAA biosynthesis pathway that control the growth of tillering buds in *P. juncea*.

In addition, we inferred that DT may accumulate more CTK relative to ST, mainly as a consequence of two DEGs encoding *IPT* and *CKX*. CTK is mainly synthesized in the root and transported upward in the xylem through transpiration, which can relieve the apical dominance caused by IAA, and affect the growth and development of tillers and the number of tillers by promoting the germination and elongation of tiller buds [62]. CTK is a direct factor regulating the growth of plant lateral buds, which directly enters the lateral buds to regulate their growth. Tillering is also promoted by CTK, and multiple tiller number can be formed by overexpression of *IPT* and low expression of *CKX*. Therefore, overexpression of *IPT* and low expression of *CKX* are a clear way to increase CTK levels and yields by increasing tiller number in *P. juncea* [63]. Thus, we suggest that the tillering ability of *P. juncea* may be determined by co-regulation associated with a variety of phytohormone. Recently, it has been reported that SLs can destroy the polar transport of IAA through cross response with IAA, so as to inhibit the growth of buds. IAA and CTK have antagonistic effects on the growth of buds, thus affecting the growth of lateral buds [23, 25]. Because the process of regulating tiller bud growth by plant hormones is complex, tillering is regulated by the common coordination of multiple phytohormone instead of a single hormone [26].

Nitrogen has an important effect on the development of plant lateral branches, which can affect the development of lateral branches by affecting the synthesis and transportation of IAA, CTK and SLs. Nitrogen deficiency will lead to the enhancement of IAA signal, the increase of SL biosynthesis and the decrease of lateral branches [62]. A great number of researches analysis indicated that nitrogen, phosphorus, potassium, boron, zinc and arsenic participate in and affect the growth of plant tillering [64], but the nutrient supply of the two genotypes was the same in this experiment. These results show that there might be a higher nitrogen utilization efficiency in DT, which may lead to the observed high tiller phenotype. Furthermore, in this research, KEGG analysis revealed that “Photosynthesis”, “Carbon metabolism” and “Biosynthesis of amino acids” were significantly enriched in the DT and ST comparison. The quality of plant nitrogen nutrition directly affects photosynthetic rate, growth and development, and ultimately affects yield and light utilization efficiency. Therefore, we infer that the different nitrogen utilization efficiencies in DT and ST may be partly due to the difference in photosynthetic efficiency. A number of researches have shown that most of the energy generated by photosynthesis and its intermediates were used for carbon and nitrogen metabolism, and the energy competition between plant nitrogen and carbon metabolism could be coordinately by increase photosynthetic efficiency [65], which indicates that DT has higher photosynthetic efficiency and can coordinate nitrogen and carbon metabolism to sustain rapid growth.

In our study, WGCNA analysis found eight distinct modules with significant correlation values (Fig. 6b), and six of the eight distinct modules were involved in phenylpropanoid biosynthesis. One of the branch pathways of phenylpropane biosynthesis is lignin. Lignin mainly accumulates in the secondary cell wall of plants, provides mechanical support for plants, and participates in the formation of ducts and the transportation of water and mineral elements. In addition, lignin is also involved in anther development, resistance to pathogen invasion, resistance to herbivore feeding, and resistance to abiotic stress etc. Compared with sparse-tillering *P. juncea*, the expression of the DEGs participated in the lignin biosynthesis pathway was downregulated in dense tillering of *P. juncea*. In wheat previous reseaches revealed that the cell wall of plant containing tiller inhibition gene (*Tin*) were thicker and the degree of lignification than freetillering NILs [66]. In sugarcane, RNAi inhibition of the *4CL* gene increased tiller numbers and significantly decreased lignin content [67]. When Ward [68] studied the tillering buds of Arabidopsis, he found that *4CL* gene was only expressed in xylem and interfascicular regions, and promoted the expression of auxin resistance 1 gene (*AXR1*), so it would inhibit the formation of tillering buds. Based on these results, the lignin content of *P. juncea* might be lower in dense-tillering than in sparse-tillering process.

### Screening of stable internal reference genes of *P. juncea*

As one of the rare wheat-relative species, *P. juncea* contains abundant resistance gene resources [69]. With the increasing research of *P. juncea*, the research of gene expression regulation has become more and more important. The quantitative measurement of gene expression requires appropriate reference genes. qRT-PCR technology can easily and accurately perform absolute and relative quantification of target gene expression [70]. Therefore, it is an urgent need to determine the stable reference genes of *P. juncea*. Gene expression analysis plays an indispensable role in the study of gene function and molecular mechanism of various organisms. The quantitative results of qRT-PCR are affected by some factors. For ensuring the accuracy and reliability of experimental quantitative results, one or more suitable reference genes are usually selected to correct and standardize the experimental data. Therefore, the selection of stable and reliable reference genes is the primary prerequisite of qRT-PCR.

High-throughput sequencing technology represented by RNA-Seq can conduct an overall level study on the gene expression of individual organisms under different treatments [71]. This technique has been widely used in plant genetic research to select stably expressed reference genes for qRT-PCR in rice [72], *Medicago sativa* [73] and *Lolium perenne* L. [74]. However, the reference genes screened under certain treatment conditions are not necessarily applicable to other biological samples or treatment conditions [75]. For example, the *ef1α*、*TUB*, *CYP* (cyclophilin) and *EIF4A* (eukaryotic initiation factor 4a) were the most stable under abiotic stress (temperature, salt and PEG) and in different tissues (roots, stems and leaves) of maize, while *ACT2* was less stable [76]. By contrast, during the development of maize grain, the stability of internal reference genes *TUB* and *ACT* was the best [77]. In our research, we utilized the RNA-seq dataset generated from *P. juncea*, in which 12 candidate reference genes were selected for further evaluation. These results established a set of stably expressed reference genes for *P. juncea.*

Five software (geNorm, NormFinder, Bestkeeper, DeltaCt and RefFinder) were used for examining the stability of the 12 candidate reference genes under various different tiller number materials in *P. juncea*. There were obvious differences in the gene stability rankings generated by the calculation methods of these software. For example, *Actin97* was ranked first by GeNorm, whereas it was ranked third by RefFinder. *Actin1* was ranked first in NormFinder and deltact. Actin gene is not only a protein, but also a relatively conservative housekeeping gene. In the screening of reference genes of most other species, actin was the most stable and suitable for reference gene. Despite these divergences, based on the five calculation methods, *Actin1* and *GAPDH* were considered the two most stable reference genes among all samples. This study result was different from that of Shen et al. [78], whose previous study was on the selection of reference genes of *Psathyrostachys huashanica*, an endangered species of same genus. That study revealed that *18S rRNA* and *UBC* were the most stable reference genes. The difference in gene expression stability between two species is likely to be the result of using different statistical algorithms to calculate the stability, on the one hand, and the result of different selected tissues and organs on the other hand. Moreover, our study is consistent with the previous reference gene testing work, indicating that *Actin* was determined to be the most stable reference gene. For instance, research on *Dactylis glomerata* L. under different abiotic treatment conditions [34], *Medicago sativa* L. under drought treatments [79] and *Leymus chinensis* [24] all reached the same conclusion. *GAPDH* is glyceraldehyde-3-phosphate dehydrogenase, which is an enzyme in glycolysis. It is widely distributed in cells in various tissues [80]. *GAPDH* was the most stable reference gene identified in *Stipagrostis pennata* under the various plant tissues at different development stages and under drought stress [81]. In addition, our research results showed that α*TUB* and *EF-1*α had stable expression in all experimental.

In summary, in the tissue of tiller nodes of *P. juncea* with different tiller numbers, the screened candidate reference gene *Actin* had the best stability, which shows that it can provide reference for the collection, breeding and genetic improvement of high-quality germplasm of *P. juncea* in the future.

## Conclusion

In this research, the tillering mechanism of perennial grass *P. juncea* was expounded by transcriptome analysis. We demonstrated that dense-tillering genotypes may be distinguished by their low expression patterns of genes involved in SL, IAA, and high expression patterns of genes involved in CTK biosynthesis at the tillering stage. Furthermore, we found that nitrogen metabolism and lignin biosynthesis can also affect the number of tillers. In the tissue of tiller nodes of *P. juncea* with different tiller numbers, the screened candidate reference gene *Actin1* had the best stability. The candidate genes revealed in our study are involved in the regulation of tillering of perennial gramineous forages. These genes provide breeding resources for the establishment of high-yield and high-quality forage grasses, and will also provide a theoretical basis for the molecular breeding of *P. juncea* in the future.

## Methods

### Plant materials

*P. juncea* plant materials used in this study were mainly came from two national plant germplasm organizations. Sixteen accessions of *P. juncea* were provided by the National Plant Germplasm Resources Conservation System of United States (NPGS). One accession was provided by the National Medium-term Gene Bank for Forage Germplasm of China. Totally seventeen accessions were used based on field tillering phenotypic evaluation. (Additional file 12: Table S5).

### Tiller related phenotypic traits measurements

Tillering traits were measured on the materials of 60 individual plants in two samples at two locations for three consecutive years. The traits measured included: natural height (cm), absolute height (cm), basal cluster diameter (cm), nutritional tiller number, tiller angle, leaf length (cm), leaf width (cm), canopy width (cm). All traits were measured with five repetitions per plant. Normality tests on sample phenotypic traits and variance analysis were conducted using Origin 2019b (MicroCal, USA). The ploidy of *P. juncea* was identified by Beckman Coulter (Beckman Kurt Co., Ltd., USA) flow cytometry, and the data were analyzed by Cyt Expert (Beckman Kurt Co., Ltd).

### RNA extraction, cDNA library construction and sequencing

Thirty dense tillering (DT) plants and another thirty sparse tillering (ST) plants were selected from the same seventeen accessions. The RNA extraction, cDNA library construction and sequencing procedures were described in our previous publication [82].

### De novo transcriptome assembly, gene annotation and DEGs analysis

Raw data in fastqc (http://www.bioinformatics.babraham.ac.uk/projects/fastqc/) format was first processed by an internal perl script. Before assembly, remove the low quality of the original sequencing data, joint contamination (including joint reads) and sequencing adapters in reads and then obtain clean reads to ensure the reliability of the results. After high-quality sequencing data is obtained, sequence assembly is carried out. Clean reads were de novo assembled using Trinity platform (http://trinityrnaseq.sourceforge.net/) [83]. The assembled transcripts were clustered twice by tgicl to remove redundancy, and finally unigenes were obtained. Then, the quality of the transcriptome sequencing library was evaluated. All unigene sequences were compared with NR (NCBI non-redundant protein sequences, http://www.ncbi.nlm.nih.gov), Swiss-Prot (A manually annotated and reviewed protein sequence database, http://www.uniprot.org/), GO (Gene Ontology, http://www.geneontology.org/), KOG/COG (Clusters of Orthologous Groups of proteins, http://www.ncbi.nlm.nih.gov/KOG/), Pfam (Protein family, http://pfam.xfam.org/), KEGG (Kyoto Encyclopedia of Genes and Genomes, http://www.genome.jp/kegg) databases using BLAST (http://www.blast2go.com/) [84]. Finally, annotation information of unigene is obtained.

To identify DEGs between dense and sparse tiller samples, the FPKM [85] (Fragments Per Kilobase of transcript per Million mapped reads) method was used to analyze gene expression levels in transcriptome sequencing data. To calculate the significance of differences in gene expression levels, FDR (false discovery rate) was used as a key indicator for DEGs screening. Differential expression analysis between the two genotype was performed using the DESeq2_EBSeq R package (1.16.1) *P* value < 0.05 and |log_2_ (FC)| ≥ 1.0 (Fold Change, FC), as identified by DESeq2_EBSeq, were assigned as the thresholds for significantly differential expression [86]. Next, all DEGs were analyzed for GO and KEGG enrichment, and the significant enrichment of GO terms and KEGG pathways was analyzed with *P* ≤ 0.05 as the threshold. A total of 7935 genes with FC ≥ 1 and FPKM > 0 in at least two samples were selected to conduct the WGCNA. The biweight-mid correlation of WGCNA package in R (v3.3.0) [87] is used to calculate the gene correlation, and the pickSoftThreshold function is used to calculate the soft threshold, and the threshold is determined to be five. The core genes were screened according to the correlation index value between genes and samples in the module. The R package was used to convert the adjacency matrix into a topological overlap matrix, and the hclust function was used to perform hierarchical clustering of the matrix [88].

### Reference gene selection for qRT-PCR and primer design

Using RNA-seq data from *P. juncea* with different tiller numbers, we chose those with high expression level genes (mostly, FPKM > 10) [78]. Finally, twelve genes were selected as candidate reference genes. Primer design for qRT-PCR was done with Primer 5.0 (Premier, Canada) according to the following parameters: amplicon product size set as 80–150 bp, primer length was 18–24 bp, melting temperature (Tm) was 50–60 °C and GC content was 40–70% (Additional file 8). The PCR amplification efficiency of each gene was assessed with an qRT-PCR standard curve, following a four-fold dilution of cDNAs (1, 1/5, 1/25, 1/625, 1/3125). Expression levels of the 12 reference genes in all samples were determined by their cycle threshold values (Ct). Five softwares (geNorm [89], NormFinder [90], Bestkeeper [91], the Delta CT method [92] and RefFinder [93]) were utilized for the calculation of candidate reference gene stabilities. Relative gene expression was quantified based on the the 2^-△△CT^ method.

### Quantitative real-time PCR (qRT-PCR) analysis

To validate the RNA-Seq expression data, ten selected DEGs involved in tillering traits were determined by one-step qRT-PCR. cDNA was synthesized from the total RNA using the HiFiScript cDNA Synthesis reagent Kit (Cowin Bio, China). The experiment was performed on an Applied Biosystems 7500 (USA) fluorescence quantitative PCR instrument using UltraSYBR Mixture (Cowin Bio). The primers for the candidate genes were designed by Primer 5.0 (Premier, Canada) and the reference gene was actin (Additional file 11 [4],). The 2^-△△CT^ method was used to evaluate the relative gene expression levels of ten DEGs [92]. Each sample included three replicates. All data were statistically analyzed using Origin2019b, and presented as the mean ± SD.

## Supplementary Information

**Additional file 1: Table S1.** Clean data and mapped reads compared to unigene of each sample.

**Additional file 2: Fig. S1.** Simulation diagram of transcriptome sequencing data saturation. The saturation curve is drawn by dividing the mapped reads into 100 equal parts, gradually increasing the number of genes detected by data viewing. The abscissa is the number of reads (in 10^6^) and the ordinate is the number of genes detected (in 10^3^).

**Additional file 3: Fig. S2.** The Pearson correlation and principal component analysis (PCA) based on all expressed genes. a, Pearson correlation. b, principal component analysis (PCA).

**Additional file 4.**

**Additional file 5.**

**Additional file 6.**

**Additional file 7.**

**Additional file 8: Table S2.** Primer sequences to amplify reference genes.

**Additional file 9: Fig. S3**. Analysis results of the candidate reference genes calculated. a, GeNorm. b, NormFinder. c, BestKeeper. d, Delta Ct.

**Additional file 10: Table S3.** Comparison of different parameters of the candidate reference genes were calculated using BestKeeper.

**Additional file 11: Table S4.** The primers used in qRT-PCR for validation of DEGs.

**Additional file 12: Table S5.** *Psathrostachys juncea* germplasm and sources.

## Data Availability

The raw sequencing reads of transcriptome data in this study are available in Sequence Read Archive (SRA) database (accession number PRJNA789128). The addresses are as follows: (https://www.ncbi.nlm.nih.gov/Traces/study/?acc=PRJNA789128).

## Abbreviations

DEGs: Differentially expressed genes
Nr: NCBI nonredundant protein
Nt: Nucleotides
KOG: Clusters of Orthologous Groups of proteins
GO: Gene Ontology
KEGG: Kyoto Encyclopedia of Genes and Genomes
Swiss-Prot: A manually annotated and reviewed protein sequence database
qRT-PCR: Quantitative Real-time PCR
PCA: Principal component analysis
IAA: Auxin
GA: Gibberellic acid
SLs: Strigolactones
CTK: Cytokinin
TB1: Teosinte Branched 1
TAC1: Tiller Angle Control 1
MOC1: Monoculm 1
CKX9: Cytokinin oxidase/ dehydrogenase 9
D27: Dwarf 27
D53: Dwarf 53
IPT: Isopentenyl transferase
GGPP: Geranylgeranyl pyrophosphate
crtB: 15-cis-phytoene synthase
lcyB: lycopene beta-cyclase
ZDS: Zeta-carotene desaturase
Z-ISO: Zeta-carotene isomerase
PDS: 15-cis-phytoene desaturase
CCD7: 9-cis-beta-carotene 9′,10′-cleaving dioxygenase
CCD8: Carotenoid cleavage dioxygenase 8
ZEP: Zeaxanthin epoxidase
LUT5: Beta-ring hydroxylase
VDE: Violaxanthin de-epoxidase
NSY: Neoxanthin synthase
NCED: 9-cis-epoxycarotenoid dioxygenase
ABAH: (+)-abscisic acid 8′-hydroxylase
ABA: Xanthoxin dehydrogenase
IL4I: L-amino-acid oxidase
YUCCA: Indole-3-pyruvate monooxygenase
ipdC: Indolepyruvate decarboxylase
ALDH: Aldehyde dehydrogenase (NAD+)
ZR: Zeatin riboside
DMAPP: Dimethylallyl diphosphate
iPTP: Isopentenyl adenosine-5 ‘- triphosphate
iPDP: Isopentenyl adenosine-5′ – diphosphate
iPMP: Isopentenyl adenosine-5 ‘- monophosphate
Nrt: Nitrate/nitrite transporter
NH_3_: Ammonia
NirA: Ferredoxin-nitrite reductase
GLUL: Glutamine synthetase
Nr: Nitrate reductase
GLU: Glutamate synthase
GLUD: Glutamate dehydrogenase (NAD(P)+)
C4H: Trans-cinnamate 4-monooxygenase
PAL: Phenylalanine ammonia-lyase
4CL: 4-coumarate--CoA ligase
CAD: Cinnamyl-alcohol dehydrogenase
CCR: Cinnamoyl-CoA reductase
HCT: Shikimate O-hydroxycinnamoyltransferase
COMT: Caffeic acid 3-O-methyltransferase
C3’H: 5-O-(4-coumaroyl)-D-quinate 3′-monooxygenase
*PROG1*: Promote Growth 1
*MAX3*: *More axial branching 3*
*AXR1*: Auxin resistant1
*EIF4A*: *E*ukaryotic initiation factor 4a
DT: Dense tillers
ST: Sparse tillers
