# Supplementary material for: Analysis of controlling genes for tiller growth of Psathyrostachys juncea based on transcriptome sequencing technology
Source: BMC Plant Biol. 2022 Sep 23;22:456. doi: 10.1186/s12870-022-03837-w (PMC9502641; doi:10.1186/s12870-022-03837-w)
Supplement: Supplementary file 1 — Additional file 1: Table S1. Clean data and mapped reads compared to unigene of each sample. [file 12870_2022_3837_MOESM1_ESM.docx]

**Table S1.** Clean data and mapped reads compared to unigene of each sample

| **Libaries** | **Clean_reads** | **Clean_bases** | **Mapped_reads** | **Mapped_ratio** | **Q20** | **Q30** | **GC_pct** |
| --- | --- | --- | --- | --- | --- | --- | --- |
| DT1 | 21,955,941 | 6,561,534,704 | 15,996,115 | 72.86% | 96.79% | 91.98% | 53.42% |
| DT2 | 21,958,143 | 6,568,292,766 | 16,030,200 | 73.00% | 97.07% | 92.60% | 54.22% |
| DT3 | 22,484,341 | 6,722,840,882 | 16,367,483 | 72.80% | 97.01% | 92.42% | 54.25% |
| ST1 | 21,903,634 | 6,545,859,214 | 15,973,804 | 72.93% | 97.04% | 92.55% | 54.13% |
| ST2 | 21,043,578 | 6,283,820,298 | 15,330,527 | 72.85% | 96.93% | 92.32% | 54.13% |
| ST3 | 20,515,330 | 6,129,587,102 | 15,038,054 | 73.30% | 96.95% | 92.33% | 53.99% |
| Total | 129,860,967 | 38,811,934,966 | 94,736,183 | - | - | - | - |
