# Supplementary material for: Analysis of controlling genes for tiller growth of Psathyrostachys juncea based on transcriptome sequencing technology
Source: BMC Plant Biol. 2022 Sep 23;22:456. doi: 10.1186/s12870-022-03837-w (PMC9502641; doi:10.1186/s12870-022-03837-w)
Supplement: Supplementary file 2 — Additional file 2: Fig. S1. Simulation diagram of transcriptome sequencing data saturation. The saturation curve is drawn by dividing the mapped reads into 100 equal parts, gradually increasing the number of genes detected by data viewing. The abscissa is the number of reads (in 106) and the ordinate is the number of genes detected (in 103). [file 12870_2022_3837_MOESM2_ESM.docx]

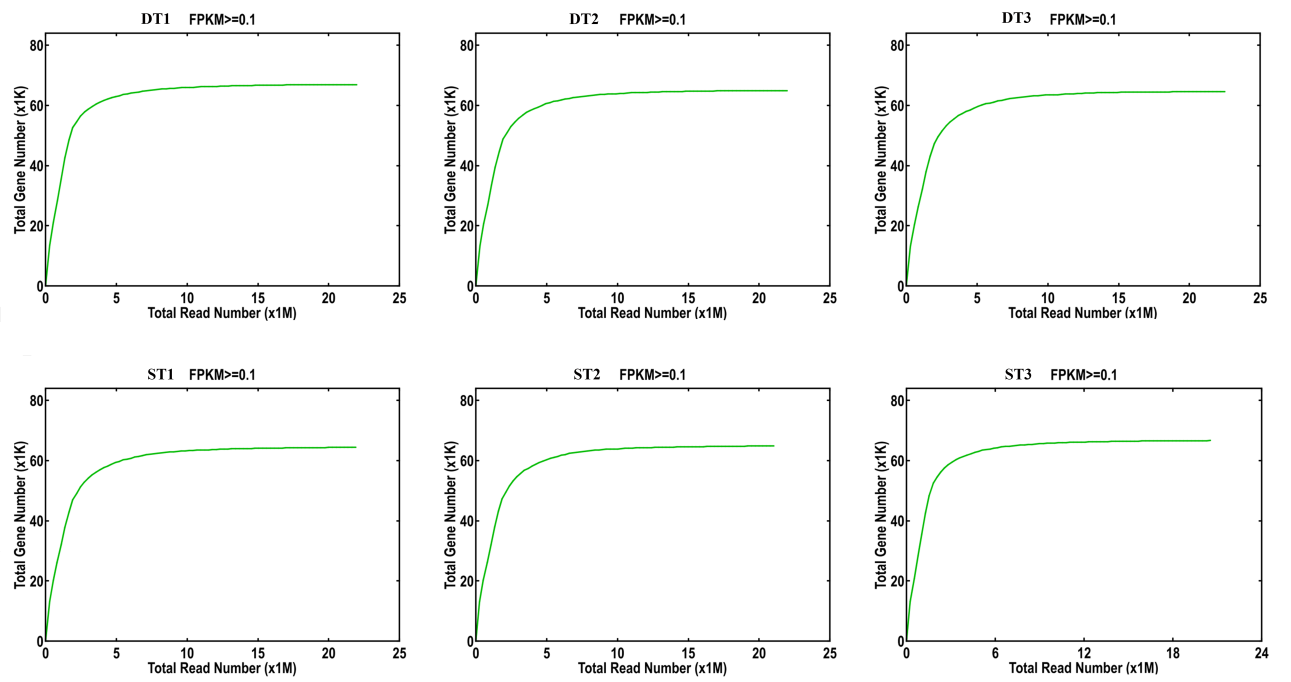


**Figure S1.** Simulation diagram of transcriptome sequencing data saturation. The saturation curve is drawn by dividing the mapped reads into 100 equal parts, gradually increasing the number of genes detected by data viewing. The abscissa is the number of reads (in 10^6^) and the ordinate is the number of genes detected (in 10^3^).
