# Supplementary material for: Analysis of controlling genes for tiller growth of Psathyrostachys juncea based on transcriptome sequencing technology
Source: BMC Plant Biol. 2022 Sep 23;22:456. doi: 10.1186/s12870-022-03837-w (PMC9502641; doi:10.1186/s12870-022-03837-w)
Supplement: Supplementary file 3 — Additional file 3: Fig. S2. The Pearson correlation and principal component analysis (PCA) based on all expressed genes. a, Pearson correlation. b, principal component analysis (PCA). [file 12870_2022_3837_MOESM3_ESM.docx]

**
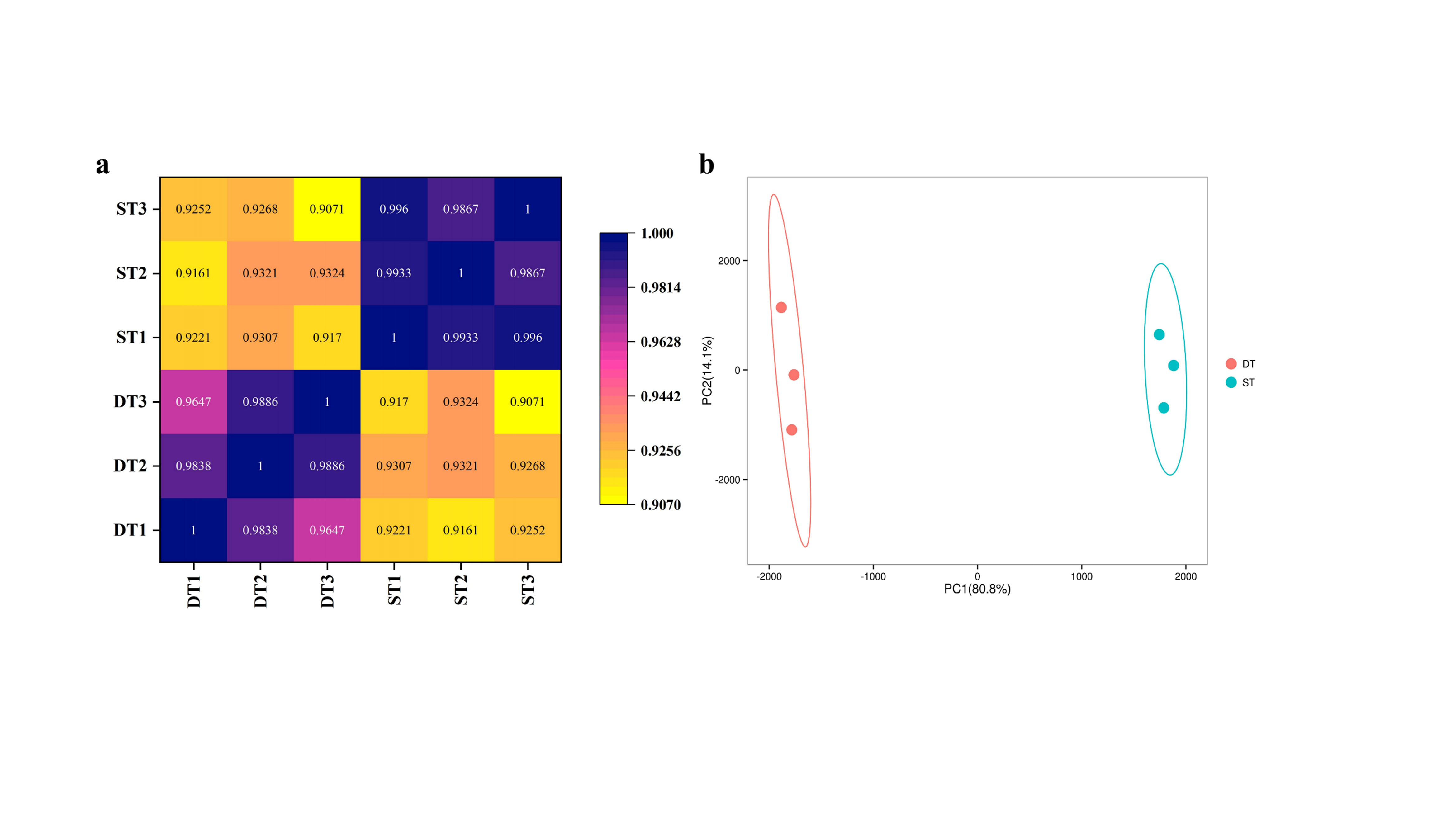
Figure S2.** The Pearson correlation and principal component analysis (PCA) based on all expressed genes. **a,** Pearson correlation. **b,** principal component analysis (PCA).
