# Supplementary material for: Analysis of controlling genes for tiller growth of Psathyrostachys juncea based on transcriptome sequencing technology
Source: BMC Plant Biol. 2022 Sep 23;22:456. doi: 10.1186/s12870-022-03837-w (PMC9502641; doi:10.1186/s12870-022-03837-w)
Supplement: Supplementary file 8 — Additional file 8: Table S2. Primer sequences to amplify reference genes. [file 12870_2022_3837_MOESM8_ESM.docx]

**Table S2.** Primer sequences to amplify reference genes

| **Gene name** | **Description** | **Forward primer(5'~3')** | **Reverse primer(5'~3')** | **Annealing temperature (◦C)** |
| --- | --- | --- | --- | --- |
| *EF-1α* | eukaryotic elongation factor 1 alpha | AAATCGTGGCGTTGCTTGAC | ATCCAGCGTAACCTGCACAA | 55.4 |
| *18S rRNA* | 18S rRNA | TCAATAACCACCCCACCAGC | CCAGGGCCGTTCTACAGTTT | 57.4 |
| *UBC2* | ubiquitin-conjugating enzyme E2–2 | GAACAAGCGCGAGTACAACC | TTGCAGGATCTGGCGACATT | 56.4 |
| *UBC28* | ubiquitin-conjugating enzyme E2–28 | TGGTGCGGAAAGAGACCTTC | TGAGGACATGTTCCACTGGC | 57.4 |
| *UBC17* | ubiquitin-conjugating enzyme E2–17 | ACACGCGACCGACAAGTAAT | CTGGACGCAGAAGTATGCCA | 56.4 |
| *αTUB* | alpha tubulin-2A | ACCAAGAGCAAGACAGGACG | GGTTGTTAAGCCATCGGTGC | 57.4 |
| *βTUB* | beta tubulin | GCGGGGGAATGGAATGAGAT | GCACACTAAAGCTTGCCACC | 57.4 |
| *Actin1* | actin 1 | TTCTTCATTGCCTGGTCCCC | TCTTGCTTGATGCGGTCCTT | 59.5 |
| *Actin97* | actin 97 | TGACCATCAGGGAGCTCGTA | AGCCGAGCGGGAAATTGTAA | 56.4 |
| *GAPDH* | Glyceraldehyde-3-phosphate dehydrogenase | CCGGTGGACTCAACAACGTA | GGACAGTGGAAGCACCATGA | 57.4 |
| *UBI* | polyubiquitin | TTGCCAGCAAAGATGATGCG | GCCAATCTTCGTGAAGACGC | 56.4 |
| *CYP* | Cytochrome P450 | GCACATGAAGTCGGGGATCA | TGATGGAGCTGTTCGCCAAG | 57.4 |
