# Supplementary material for: Analysis of controlling genes for tiller growth of Psathyrostachys juncea based on transcriptome sequencing technology
Source: BMC Plant Biol. 2022 Sep 23;22:456. doi: 10.1186/s12870-022-03837-w (PMC9502641; doi:10.1186/s12870-022-03837-w)
Supplement: Supplementary file 9 — Additional file 9: Fig. S3. Analysis results of the candidate reference genes calculated. a, GeNorm. b, NormFinder. c, BestKeeper. d, Delta Ct. [file 12870_2022_3837_MOESM9_ESM.docx]

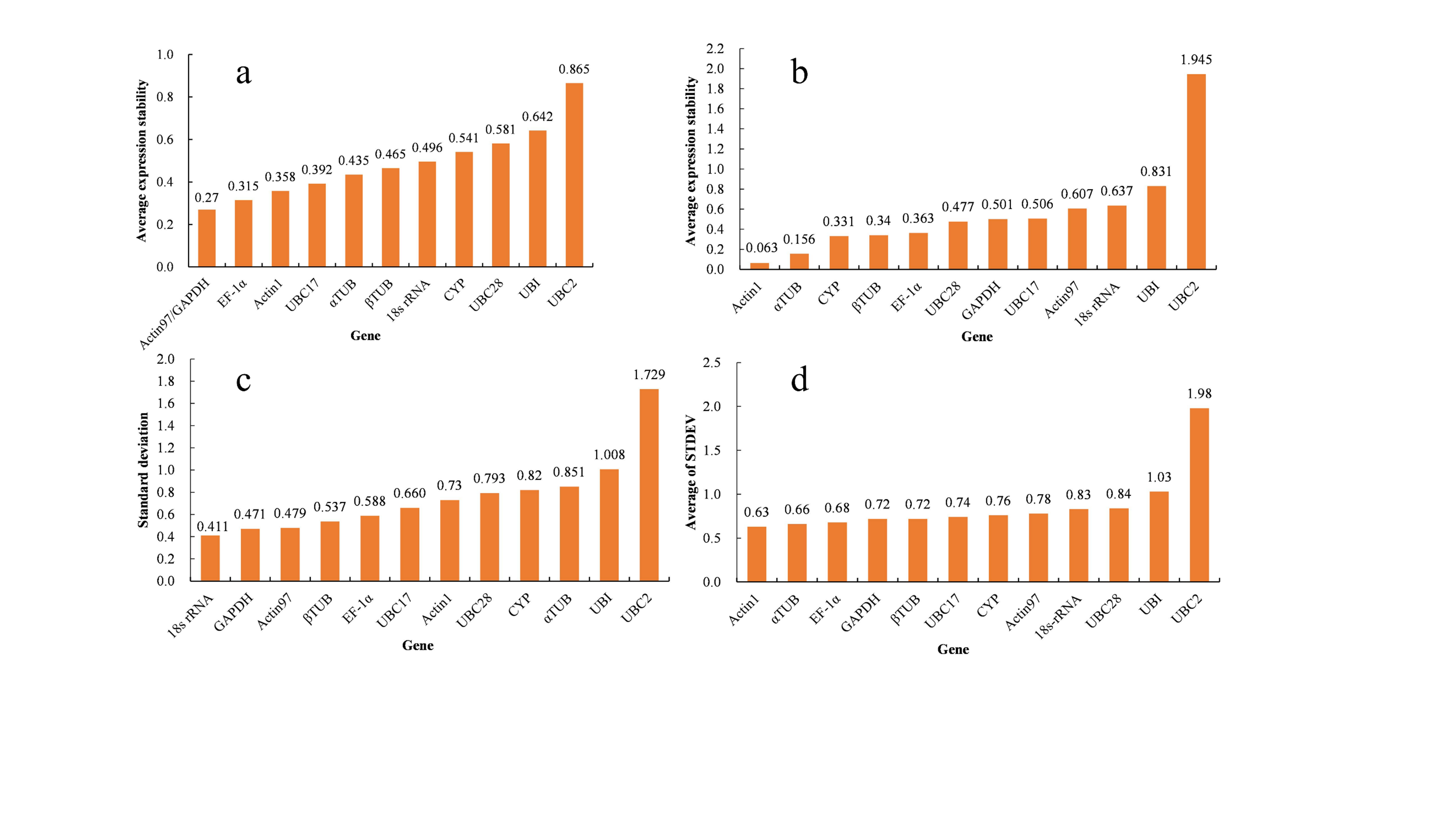


**Figure S3**. Analysis results of the candidate reference genes calculated. **a,** GeNorm. **b,** NormFinder. **c,** BestKeeper. **d,** Delta Ct.
