# Supplementary material for: Analysis of controlling genes for tiller growth of Psathyrostachys juncea based on transcriptome sequencing technology
Source: BMC Plant Biol. 2022 Sep 23;22:456. doi: 10.1186/s12870-022-03837-w (PMC9502641; doi:10.1186/s12870-022-03837-w)
Supplement: Supplementary file 10 — Additional file 10: Table S3. Comparison of different parameters of the candidate reference genes were calculated using BestKeeper. [file 12870_2022_3837_MOESM10_ESM.docx]

**Table S3.** Comparison of different parameters of the candidate reference genes were calculated using BestKeeper

| **Gene** | **Mean** | **Maximum** | **Minimum** | **Standard deviation (SD)** | **Coefficient variable (CV)%** | **Correlation coefficient variable (*r*)** | ***P* value** |
| --- | --- | --- | --- | --- | --- | --- | --- |
| *EF-1α* | 31.02 | 32.07 | 30.14 | 0.59 | 1.89 | 0.911 | 0.012 |
| *18S rRNA* | 28.16 | 28.66 | 27.4 | 0.41 | 1.46 | 0.784 | 0.065 |
| *UBC2* | 29.65 | 34.93 | 27.93 | 1.73 | 5.81 | 0.911 | 0.012 |
| *UBC28* | 30.22 | 32.62 | 29.25 | 0.79 | 2.62 | 0.924 | 0.008 |
| *UBC17* | 30.31 | 31 | 29.09 | 0.66 | 2.18 | 0.845 | 0.034 |
| *αTUB* | 33.89 | 34.5 | 32.93 | 0.85 | 2.51 | 0.984 | 0.001 |
| *βTUB* | 31.75 | 33.37 | 30.93 | 0.54 | 1.69 | 0.908 | 0.012 |
| *Actin1* | 25.49 | 26.91 | 24.3 | 0.73 | 2.86 | 0.975 | 0.001 |
| *Actin97* | 23.89 | 24.39 | 23.18 | 0.48 | 2 | 0.787 | 0.063 |
| *GAPDH* | 26.18 | 26.75 | 25.1 | 0.47 | 1.8 | 0.865 | 0.026 |
| *UBI* | 27.84 | 28.91 | 26.09 | 1.01 | 3.62 | 0.763 | 0.078 |
| *CYP* | 30.15 | 32.21 | 28.55 | 0.82 | 2.72 | 0.976 | 0.001 |
