# Supplementary material for: Analysis of controlling genes for tiller growth of Psathyrostachys juncea based on transcriptome sequencing technology
Source: BMC Plant Biol. 2022 Sep 23;22:456. doi: 10.1186/s12870-022-03837-w (PMC9502641; doi:10.1186/s12870-022-03837-w)
Supplement: Supplementary file 11 — Additional file 11: Table S4. The primers used in qRT-PCR for validation of DEGs. [file 12870_2022_3837_MOESM11_ESM.docx]

| **Gene ID** | **Gene name** | **Forward primer(5'~3')** | **Reverse primer(5'~3')** |
| --- | --- | --- | --- |
| Unigene_065650 | *IPT* | GCTGCGAGTCATCGAGAGAT | AGAACGCTTCCCGAACTAGC |
| Unigene_071842 | *CKX4* | CGCTCAAATGTCTCCCACCT | ATGAAGCAGTACCTAGCGCC |
| Unigene_026272 | *CKX5* | GATCGACCAACTTGCCTTGC | CTTGCGAAAGATCGCGTGAG |
| Unigene_142326 | *D27* | AGTTTTCGGCTGTCCATCGT | TTTTCCTAAACCGGCCCCTC |
| Unigene_083877 | *CCD7* | CCTTCGCTATGCTGTCGAGT | CAACTGTCCGTCTCTGCTGT |
| Unigene_136214 | *CCD8* | GAAGGAACCAACCGTCGTCT | GAAGACGGCCAAGAACTGGT |
| Unigene_030280 | *YUCCA* | GACATCGCAACCGAGTACCA | TTGAGATCCATGCTCTCGCC |
| Unigene_013875 | *CCR* | CTTCGCGTAGCAGTACCAGT | CCATCCCGGCCCTATATACC |
| Unigene_020652 | *4CL* | AATACATAGCGGAGCGAGGC | GTCATCCTCTCCCGGACAAC |
| Unigene_169973 | *PAL* | GCCGCTATCATGGAGCACAT | ATGGGTCAAGCTCACCAAGC |
|  | *Actin* | TGGTATGGAAGCTGCTGGAA | TCAGCAATACCCGGGAACAT |

**Table S4.** The primers used in qRT-PCR for validation of DEGs
