# Supplementary material for: Analysis of controlling genes for tiller growth of Psathyrostachys juncea based on transcriptome sequencing technology
Source: BMC Plant Biol. 2022 Sep 23;22:456. doi: 10.1186/s12870-022-03837-w (PMC9502641; doi:10.1186/s12870-022-03837-w)
Supplement: Supplementary file 12 — Additional file 12: Table S5. Psathrostachys juncea germplasm and sources. [file 12870_2022_3837_MOESM12_ESM.docx]

**Table S5.** *Psathrostachys juncea* germplasm and sources

| **Order** | **Accession ID** | **Varieties name** | **Origin** | **Germplasm preservation institutions** | **Cultivation** | Number of plants used | |
| --- | --- | --- | --- | --- | --- | --- | --- |
|  |  |  |  |  |  | **Sample1** | **Sample2** |
| 1 | PI 549118 | BOZOISKY SELECT | Utah, U.S. | NPGS, U.S. | Cultivate | 2 | 2 |
| 2 | PI 595135 | X 93031 | Xin jiang,China | NPGS, U.S. | Wild | 2 | 2 |
| 3 | PI 531826 | D-3139 | China | NPGS, U.S. | Wild | 1 | 1 |
| 4 | CF 005043 | Shandan | China | National Medium term Gene Bank of Forage Germplasm, China | Cultivate | 2 | 2 |
| 5 | PI 502577 | K 40175 | Russian Federation | NPGS, U.S. | Cultivate | 2 | 2 |
| 6 | PI 565060 | AJC-534 | Russian Federation | NPGS, U.S. | Wild | 1 | 1 |
| 7 | PI 619487 | 96N-300 | Mongolia | NPGS, U.S. | Wild | 2 | 2 |
| 8 | PI 619565 | 96N-238 | Mongolia | NPGS, U.S. | Wild | 2 | 2 |
| 9 | PI 531828 | — | Idaho, U.S. | NPGS, U.S. | Wild | 2 | 2 |
| 10 | PI 531827 | — | Estonia | NPGS, U.S. | Wild | 2 | 2 |
| 11 | PI 502573 | AR-163 | Former Soviet Union | NPGS, U.S. | Cultivate | 2 | 2 |
| 12 | PI 272136 | — | Alma-Asa, Kazakhstan | NPGS, U.S. | Cultivate | 2 | 2 |
| 13 | PI 499559 | D-2697 | China | NPGS, U.S. | Wild | 2 | 2 |
| 14 | PI 565044 | DJ-3890 | Russian Federation | NPGS, U.S. | Wild | 2 | 2 |
| 15 | PI 499560 | D-2562 | China | NPGS, U.S. | Cultivate | 1 | 1 |
| 16 | PI 598610 | VIR U-0134973 | Kazakhstan | NPGS, U.S. | Wild | 1 | 1 |
| 17 | PI 665583 | KGZ-06-41-208 | Naryn, kyrgyzstan | NPGS, U.S. | Wild | 2 | 2 |
